# Single-cell RNA sequencing uncovers a neuron-like macrophage subset associated with cancer pain

**DOI:** 10.1126/sciadv.abn5535

**Published:** 2022-10-07

**Authors:** Philip Chiu-Tsun Tang, Jeff Yat-Fai Chung, Jinyue Liao, Max Kam-Kwan Chan, Alex Siu-Wing Chan, Guangyao Cheng, Chunjie Li, Xiao-Ru Huang, Calvin Sze-Hang Ng, Eric W-F Lam, Dongmei Zhang, Yi-Ping Ho, Ka-Fai To, Kam-Tong Leung, Xiaohua Jiang, Ho Ko, Tin-Lap Lee, Hui-Yao Lan, Patrick Ming-Kuen Tang

**Affiliations:** ^1^ Department of Anatomical and Cellular Pathology, State Key Laboratory of Translational Oncology, The Chinese University of Hong Kong, Hong Kong SAR.; ^2^ Department of Chemical Pathology, Faculty of Medicine, The Chinese University of Hong Kong, Hong Kong SAR.; ^3^ Department of Applied Social Sciences, The Hong Kong Polytechnic University, Hong Kong SAR.; ^4^ Department of Biomedical Engineering, The Chinese University of Hong Kong, Shatin, New Territories, Hong Kong SAR.; ^5^ Department of Head and Neck Oncology, West China Hospital of Stomatology, Sichuan University, Chengdu, Sichuan, China.; ^6^ Department of Medicine and Therapeutics, Li Ka Shing Institute of Health Sciences, The Chinese University of Hong Kong, Hong Kong SAR.; ^7^ Department of Surgery, The Chinese University of Hong Kong, Hong Kong SAR.; ^8^ State Key Laboratory of Oncology in South China, Cancer Center, Sun Yat-sen University, 651 Dongfeng East Road, Guangzhou, Guangdong Province 510060, China.; ^9^ College of Pharmacy, Jinan University, Guangzhou, China.; ^10^ Department of Paediatrics, The Chinese University of Hong Kong, Shatin, Hong Kong SAR.; ^11^ Key Laboratory for Regenerative Medicine of the Ministry of Education of China, School of Biomedical Sciences, Faculty of Medicine, The Chinese University of Hong Kong, Hong Kong SAR.; ^12^ Reproduction, Development and Endocrinology Program, School of Biomedical Sciences, The Chinese University of Hong Kong, Hong Kong SAR.; ^13^ Guangdong-Hong Kong Joint Laboratory on Immunological and Genetic Kidney Diseases, The Chinese University of Hong Kong, 999077 Hong Kong SAR.

## Abstract

Tumor innervation is a common phenomenon with unknown mechanism. Here, we discovered a direct mechanism of tumor-associated macrophage (TAM) for promoting de novo neurogenesis via a subset showing neuronal phenotypes and pain receptor expression associated with cancer-driven nocifensive behaviors. This subset is rich in lung adenocarcinoma associated with poorer prognosis. By elucidating the transcriptome dynamics of TAM with single-cell resolution, we discovered a phenomenon “macrophage to neuron-like cell transition” (MNT) for directly promoting tumoral neurogenesis, evidenced by macrophage depletion and fate-mapping study in lung carcinoma models. Encouragingly, we detected neuronal phenotypes and activities of the bone marrow–derived MNT cells (MNTs) in vitro. Adoptive transfer of MNTs into NOD/SCID mice markedly enhanced their cancer-associated nocifensive behaviors. We identified macrophage-specific Smad3 as a pivotal regulator for promoting MNT at the genomic level; its disruption effectively blocked the tumor innervation and cancer-dependent nocifensive behaviors in vivo. Thus, MNT may represent a precision therapeutic target for cancer pain.

## Teaser

Macrophage to neuron-like cell transition (MNT) is a direct mechanism and potential therapeutic target for cancer pain.

## INTRODUCTION

Cancer is still a leading cause of death worldwide. Ineffective treatments, severe side effects, drug resistance, recurrence, and metastasis are the major barriers to cancer symptom management and cure (*1*). In particular, about half of all patients diagnosed with malignancies experience chronic pain, which seriously affects their quality of life. This is especially pertinent in patients with advanced cancers of the lung, breast, prostate, or gastrointestinal tract, as two-thirds of these patients suffer from chronic pain (*2*, *3*). Globally, nearly 80% of cancer patients, including pediatric patients, have inadequate pain control (*4*). It is therefore of paramount importance to elucidate the underlying mechanisms of cancer pain to guide therapeutics development.

Cancer pain is thought to be partly caused by tissue damage and inflammation in the tumor microenvironment (TME) via unclear mechanisms (*4*). Clinically, chronic inflammation is often associated with the development and progression of cancer (*5*, *6*). It is still unclear how chronic inflammation drives cancer complications especially chronic pain (*4*). In the past few decades, neuronal plasticity has been established as a key mechanism for the development and maintenance of chronic pain (*7*, *8*). Peripheral sensitization commonly occurs after peripheral tissue injury and inflammation at the nociceptor level; it is essential for transitioning acute nociception into chronic pain (*9*, *10*). Nociceptive sensory neurons not only respond to immune signals but also directly modulate inflammation, evidenced by the finding that silencing nociceptor reduces inflammation in the allergic airway (*11*).

Increasing evidence suggests that numerous non-neuronal cell types play an important role in pain sensation including cancer cells and macrophages (*4*). An emerging role for macrophages in periphery pain regulation has recently been suggested. Accordingly, depletion of macrophages, but not of neutrophils or T cells effectively attenuates the development of incision and pathogen-induced mechanical and thermal hypersensitivity in mice (*12*). This is accompanied by the down-regulation of interleukin-1β (IL-1β) and other pro-algetic mediators at inflammatory sites (*13*). Macrophages were also found to affect analgesia by indirectly releasing anti-inflammatory mediators, such as IL-10 and specialized pro-resolution mediators (*14*, *15*). Furthermore, a recent study demonstrated that resident microglia and peripheral monocytes act synergistically to initiate hypersensitivity and promote chronic pain after peripheral nerve injury (*16*). Although the involvement of macrophages in evoked pain has been suggested by the suppressive effect of macrophage depletion on mechanical and cold hypersensitivity (*17*, *18*), the functional role and regulatory mechanism of macrophages, especially tumor-associated macrophages (TAMs), in cancer pain is still largely unknown.

Single-cell RNA sequencing (scRNA-seq) is an emerging approach to resolve cellular heterogeneity on a genome-wide scale. It can dissect the reconstitute temporal transcription networks during developmental processes or external stimuli that are often masked at a population level (*19*, *20*). The advancement of high-throughput techniques for scRNA-seq is leading to profound discoveries in biology by enhancing the identification of previously unknown cell types and therapeutic targets (*21*). Transcriptome signatures and phenotypes are highly dynamic in the TME, where the phenotypes of macrophages can be rapidly changed via transcriptional regulation because of stimulations from cancer cells (*22*).

We have successfully dissected the dynamics of macrophages under conditions of tissue inflammation and cancer by scRNA-seq (*23*–*25*) and unexpectedly uncovered a strong association of the transforming growth factor–β1 (TGF-β1)/Smad3–dependent transcriptomes of bone marrow–derived macrophages (BMDMs) with cancer and neuronal development (differentiation, generation, and morphogenesis) (*24*). Previous studies show that cancer cell secretomes promote neurogenesis for tumor innervation in prostate and pancreatic cancer (*26*, *27*). TGF-β and its canonical downstream effector Smad3 also regulate the nociceptive pain in chronic pancreatitis (*28*), suggesting an unexplored role of TAMs in the TGF-β1/Smad3–driven neurogenesis. Here, we discovered how TGF-β1/Smad3 signaling promotes tumor innervation by TAM via a direct mechanism, which may represent a previously unidentified therapeutic target for handling cancer pain in the future.

## RESULTS

### A previously unknown neuron-like TAM subset exists in experimental lung carcinoma TME

**Fig. 1.**
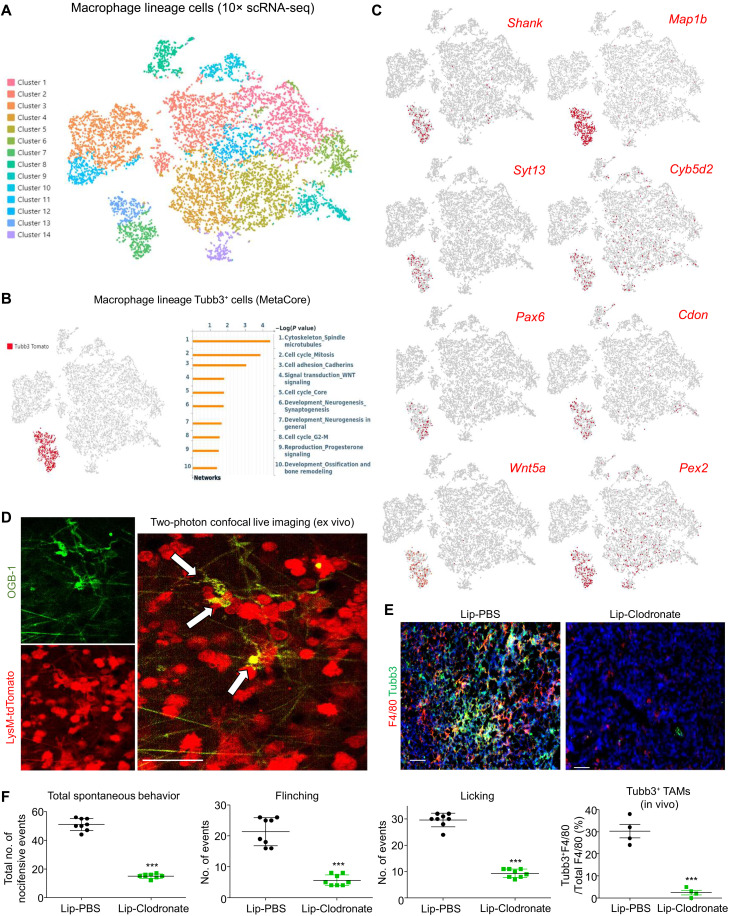
Macrophage-specific scRNA-seq reveals a previously unknown neuron-like TAM subset. (**A**) *t*-Distributed stochastic neighbor embedding (*t*-SNE) of 10×-based scRNA-seq of LysM-tdTomato^+^ macrophage lineage cells from the LLC tumors on LysM-Cre/ROSA-tdTomato mice. (**B**) GO analysis of up-regulated DEGs in the macrophage lineage–derived Tubb3^+^ cells (red cluster) reveals their association with neurogenesis as shown by GO terms “Development_Neurogenesis_Synaptogenesis” (*P* = 0.01980) and “Development_Neurogenesis_in_general” (*P* = 0.02536). (**C**) Expression level of neuronal genes in the *t*-SNE plot of mouse Tubb3^+^ TAMs. (**D**) Two-photon confocal live imaging detects calcium efflux in the LysM-tdTomato^+^ macrophage-derived neuronal-like cells (red) in the LLC tumor ex vivo, as shown by their concentrated OGB-1 (green fluorescent calcium indicator dye) intensity. (**E**) Clodronate liposomes (Lip-Clodronate)–mediated macrophage depletion significantly reduces Tubb3^+^ TAMs in LLC tumor compared to their control group [liposome phosphate-buffered saline (Lip-PBS)], as shown by immunofluorescence (*n* = 4; ***P < 0.001 versus Lip-PBS, *t* test), and (**F**) is associated with a significant reduction of spontaneous nocifensive behaviors (total counts of flinching and licking observed in 30 min) of the LLC-bearing mice on day 20 [*n* = 4 (duplicate); ***P < 0.001 versus Lip-PBS, *t* test]. Scale bars, 50 μm.

To better understand the dynamics and potential contribution of TAMs in the tumor innervation, we submitted the flow-sorted macrophage lineage cells from the tumor of LysM-Cre/ROSA-tdTomato mice (*29*) with syngeneic lung carcinoma model LLC for 10× scRNA-seq (Fig. 1A). To note, LysM expression is almost undetectable in mouse brain cells (fig. S1A). This system allows us to trace origins of the macrophage lineage cells, even when their lineage markers were lost during maturation and differentiation (*30*). We detected a TAM subset with strong expression of the neuron differentiation marker Tubb3 in the macrophage lineage cells of LLC tumor; their up-regulated differentially expressed genes (DEGs) were highly associated with neurogenesis shown by gene ontology (GO) analysis with the MetaCore bioinformatics platform (Fig. 1B and file S1) (*24*). We also observed the expression of neurogenesis-associated genes (i.e., Shank, Map1b, Syt13, Cyb5d2, Pax6, Cdon, Wnt5a, and Pex2) in the Tubb3^+^ TAMs of LLC tumors with a loss of macrophage marker expression at the transcriptional level in vivo (Fig. 1C and figs. S1B and S2).

Therefore, we attempted to explore this previously uninvestigated neuron-like subset in the TME at the protein level by imaging calcium efflux with Oregon-Green-BAPTA-1 (OGB-1) (*31*). Encouragingly, two-photon live imaging evidenced the existence of macrophage lineage (tdTomato^+^)–derived cells, with nerve fiber–like morphology (white narrow pointed) showing enhanced calcium efflux level (OGB-1^+^) in the LLC tumor ex vivo (Fig. 1D and movie S1). We demonstrated that liposome clodronate–mediated macrophage depletion canceled the formation of this neuron-like subset in the LLC tumor associated with a significant reduction of the cancer-associated nocifensive behaviors (Fig. 1, E and F) (*32*). The presence of Tubb3^+^ TAMs was highly associated with the cancer-associated nocifensive behaviors such as flinching and licking (*32*), suggesting that this previously unknown TAM subset may be capable of promoting tumoral sensation via a direct mechanism.

### The neuron-like TAM subset is associated with neurogenesis in NSCLC

**Fig. 2.**
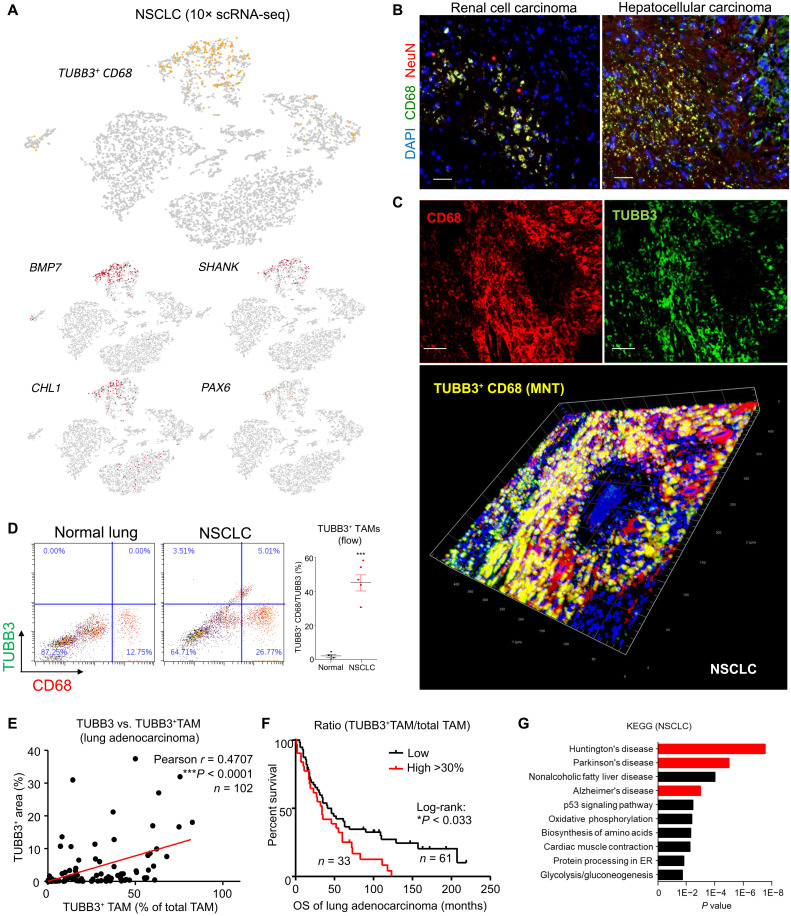
Presence of the neuron-like TAM subset in NSCLC. (**A**) Existence of the TUBB3^+^CD68 neuron-like TAM subset (yellow) and their expression levels of neuronal genes (red) in a surgical resection of human NSCLC biopsy detected by 10× scRNA-seq. (**B**) Detection of the NeuN^+^ TAM in patient biopsies of renal cell carcinoma and hepatocellular carcinoma by immunofluorescence (quantification in fig. S4B). (**C**) Confocal microscopy with z-stack scanning confirms a TUBB3^+^ TAM subset (TUBB3^+^ CD68, yellow) in NSCLC biopsy. (**D**) The levels of TUBB3^+^ TAM in tumor and normal lung tissues were quantified with flow cytometry analysis (*n* = 5; *t* test, ***P < 0.001 versus Normal). Cohort analysis of adenocarcinoma NSCLC shows significance in (**E**) correlation (% area of total TUBB3^+^ cells versus ratio of TUBB3^+^ TAMs/total CD68; ***P < 0.0001, Pearson correlation; *n* = 102) and (**F**) higher mortality of patients with high TUBB3^+^ TAM level, quantified by multiplex immunohistochemical staining (*n* = 94; *P* < 0.033, log-rank test). (**G**) The up-regulated DEGs of human TUBB3^+^ CD68^+^ TAMs are highly associated with neuronal diseases in GO analysis (Huntington’s disease, Parkinson’s disease, and Alzheimer’s disease, red). Scale bars, 50 μm.

Encouragingly, we also found this TUBB3^+^CD68 TAM subset in a 10× scRNA-seq dataset of human non–small cell lung cancer (NSCLC) biopsy with strong expression of neuronal genes including BMP7, SHANK, CHL1, and PAX6 (Fig. 2A). This previously unknown TUBB3^+^ TAM subset showed a neuron-like transcriptome signature similar to the TUBB3^+^ neuronal cells but distant from TAMs at the single-cell level and was highly associated with human neural diseases (fig. S3 and file S2). The existence of this neuron-like TAM subset was also found in human renal cell and hepatocellular carcinoma (Fig. 2B and fig. S4B) and further confirmed with NSCLC patient biopsies in large scale (*n* = 102). The previously unknown TAM subset was further visualized in NSCLC by confocal three-dimensional imaging (Fig. 2C), and their tumor-specific localization was detected by flow cytometric analysis comparing NSCLC and normal lung tissue (Fig. 2D and fig. S5A). This TUBB3^+^ TAM subset was highly present in the adenocarcinoma NSCLC and was positively correlated with the expression of neuronal differentiation marker TUBB3 in the human TME (*r* = 0.4707, *P* < 0.0001, *n* = 102; Fig. 2E and fig. S4A). To note, the abundance of the TUBB3^+^ TAMs (TUBB3^+^ CD68^+^/CD68^+^ ratio > 30%) was significantly associated with poorer survival of older NSCLC patients (age > 60; *n* = 94; *P* < 0.033, log-rank test; Fig. 2F) and their up-regulated DEGs were highly associated with neural diseases (Huntington’s disease, Parkinson’s disease, and Alzheimer’s disease; red, Fig. 2G and file S2), suggesting a potential contribution of this previously unidentified TAM subset to neurogenesis.

### Macrophage to neuron-like cell transition in TME

**Fig. 3.**
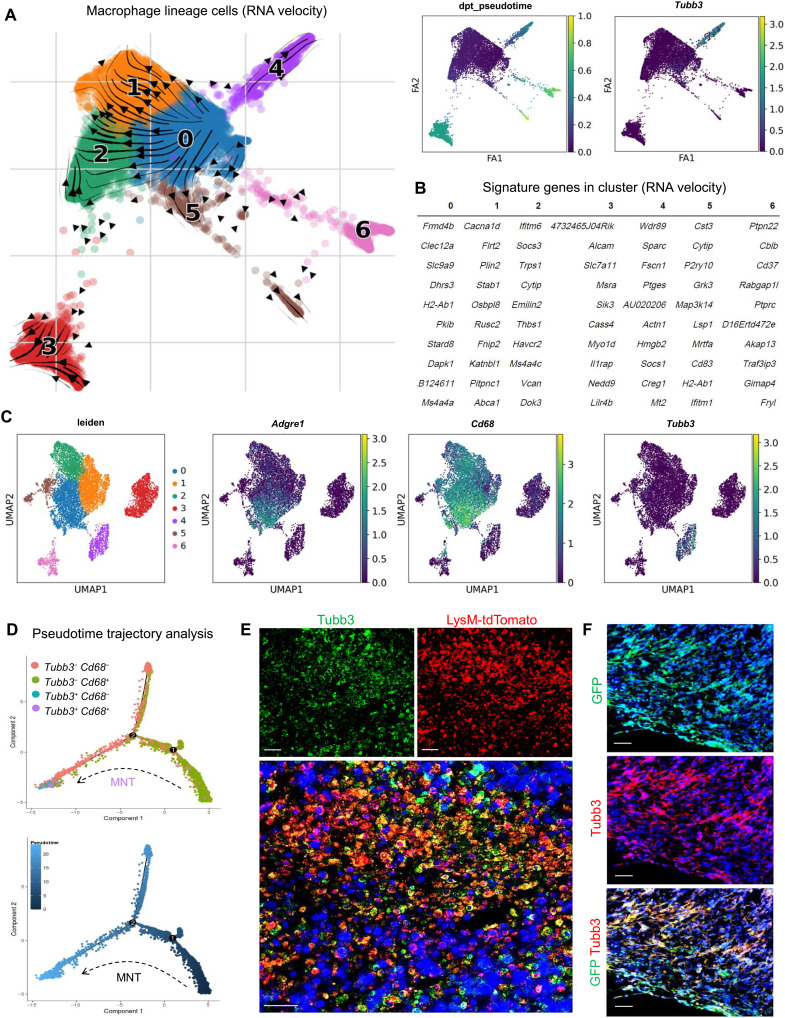
MNT in TME. (**A**) RNA velocity analysis shows a macrophage lineage–derived Tubb3^+^ population (cluster 4, purple) derived from the F4/80^+^ TAM (cluster 0, blue) in the LysM-tdTomato cell scRNA-seq dataset from Fig. 1A. (**B**) Their top expression genes show an obvious macrophage and neuronal signatures in clusters 0 and 4. (**C**) UMAP of the LysM-tdTomato cell scRNA-seq dataset from Fig. 1A, where the Tubb3-rich cluster (cluster 4, purple) is linked but is discrete from Cd68- and Adgre1 (F4/80)–rich cluster (cluster 0, blue) among seven clusters of the macrophage lineage cells in the TME. (**D**) Pseudotime analysis consistently demonstrates a complete transition from Tubb3^−^ Cd68^+^ TAM into Tubb3^+^ Cd68^−^ neuron-like cells in the TME at the transcriptional level. (**E**) Fate-mapping study evidences the existence of macrophage lineage–derived Tubb3^+^LysM-tdTomato^+^ population in the LLC tumor on day 15 (quantification in fig. S8, A and B). (**F**) Adoptive transfer of GFP^+^ BMDMs results in the production of GFP^+^ Tubb3^+^ cells in the LLC tumor on day 25 detected at the protein level by immunofluorescence (*n* = 5; quantification in fig. S8C). Scale bars, 50 μm.

To better understand the dynamics and potential contribution of this Tubb3^+^ TAM subset in tumoral neurogenesis, we elucidated the TAM-specific scRNA-seq dataset (Fig. 1A and fig. S5B) with the latest bioinformatic platform RNA velocity analysis (fig. S6), a kinetic model of RNA transcription and splicing to estimate individual cell states to allow causal inference of terminally differentiated cells from parent cells (*33*, *34*). As shown in Fig. 3A, the arrows in the diffusion map and its diffusion pseudotime demonstrated that the Tubb3^+^ cluster (cluster 4) derived from the F4/80^+^ cluster (cluster 0), which also serves as the parent cluster of whole macrophage lineage by both RNA velocity and MuTrans analytic methods (fig. S7). Moreover, a shift of macrophage (Clec12a, Dhrs3, H2-Ab1, and Stard8) to neuron-associated gene signatures (Sparc, Fscn1, Hmgb2, and Socs1; Fig. 3B) was observed in the top 10 genes of clusters 0 and 4, respectively. Consistently, a Tubb3-rich cluster (cluster 4; purple, Fig. 3C) was identified from the macrophage lineage cells of LLC tumor by Uniform Manifold Approximation and Projection (UMAP), which is adjacent to Cd68- and Adgre1 (F4/80)–rich cluster (cluster 0, blue). To examine the potential direct transition of macrophages into neuron-like cells in TME, their relationship during developmental stages was further visualized by pseudotime analysis. The macrophage lineage cells expressing CD68 and/or Tubb3 were extracted to reconstruct a putative developmental trajectory, which showed a clear path from the least (Tubb3^−^ Cd68^+^) to the most differentiated neuron-like cells (Tubb3^+^ Cd68^−^; Fig. 3D and fig. S7).

Furthermore, we observed macrophage lineage–derived Tubb3^+^ cells in the LLC tumors of the LysM-Cre/ROSA-tdTomato mice in vivo by confocal imaging (Fig. 3E), accounting for more than 90% of the total tumoral Tubb3^+^ cells detected by flow cytometric and image analysis (fig. S8, A and B). This observation was further confirmed with a LysM-independent method by the adoptive transfer of green fluorescent protein (GFP)–expressing BMDMs in vivo (Fig. 3F and fig. S8C). These findings suggested the previously unidentified phenomenon “macrophage to neuron-like cell transition” (MNT), which may serve as a previously unknown and rich source of neuron-like cells for directly promoting tumor innervation.

### MNTs show neuronal phenotypes and activities in vitro

**Fig. 4.**
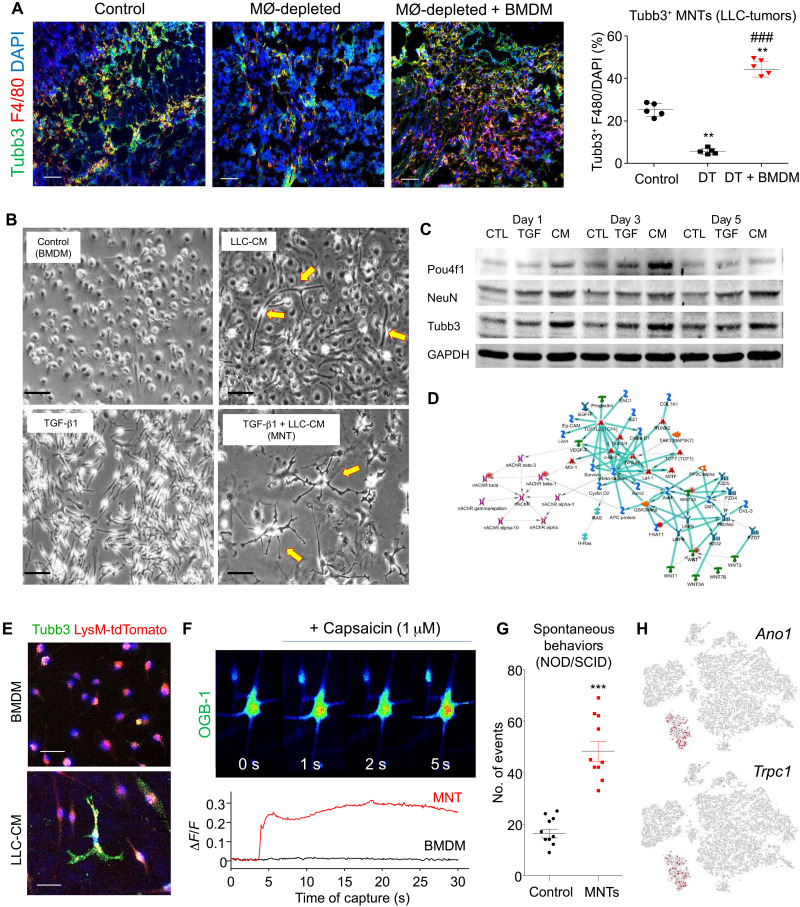
Neuronal phenotypes and activity of MNTs. (**A**) Diphtheria toxin–mediated macrophage depletion reduces Tubb3^+^F4/80^+^ MNTs in the LLC tumor of LysM/iDTR mice, which is successfully rescued by the adoptive transfer of BMDM as shown by immunofluorescence and quantification [**P < 0.01 versus Control, ^###^*P* < 0.01 versus DT, one-way analysis of variance (ANOVA); *n* = 5]. (**B**) Morphological changes of BMDMs under stimulation with TGF-β1 (5 ng/ml for 5 days), cancer cell conditioned medium LLC-CM [10% (v/v) for 7 days], and their combination (TGF-β1 for 5 days and then LLC-CM for 7 days) in vitro (quantification in fig. S8D). (**C**) The de novo expression of neuronal differentiation markers in BMDM under TGF-β1 (TGF) and LLC-CM (CM) is detected by Western blot analysis (quantification in fig. S8E; ***P < 0.001 versus Control; ^###^*P* < 0.001 versus TGF-β1, one-way ANOVA; *n* = 3). (**D**) Unbiased bioinformatic analysis reconstructs a neurotransmitter-centric gene network by using the up-regulated DEGs of MNTs (Tubb3^+^ TAM in Fig. 1B). (**E**) Strong expression of Tubb3 (green) and neuron-like dendrites is detected in the in vitro generated MNTs but not in BMDMs (quantification in fig. S11B) as shown by immunofluorescence, where (**F**) capsaicin (activator of nociceptive vanilloid receptor) (*41*) markedly induces calcium efflux of the MNTs but not BMDMs as detected by the change of OGB-1 fluorescent intensity (Δ*F*/*F*). (**G**) Local adoptive transfer of in vitro generated MNTs significantly increased spontaneous nocifensive behaviors of the LLC-bearing NOD/SCID mice compared to their LLC only control in vivo [***P < 0.001 versus Control, *t* test; *n* = 5 (duplicate)], (**H**) where MNTs in LLC tumor highly express pain mediators Ano1 and Trpc1 detected by macrophage lineage scRNA-seq dataset from Fig. 1A. Scale bars, 50 μm (A) and 25 μm (B and E).

To examine the involvement of this previously unknown TAM subset in tumor innervation, we conducted macrophage depletion in our well-established LysM-Cre/iDTR mice bearing syngeneic lung carcinoma LLC (*35*, *36*). We found a significant reduction of the Tubb3^+^ TAMs after depletion (DT group), which was successfully rescued in the LLC tumor by the adoptive transfer of BMDMs in vivo (Fig. 4A). We observed neuronal morphology in the BMDMs upon stimulation with the conditioned medium of LLC cancer cells (CM) in vitro (Fig. 4B); in contrast, fibroblast-like morphology was found under TGF-β1 stimulation as in our previous study (*24*). Unexpectedly, we found that the CM-induced neuronal morphology of BMDMs can be further enhanced by the TGF-β1 pretreatment (fig. S8D). Consistently, we detected higher expression of neuronal differentiation mediators (i.e., Tubb3, NeuN, and Pou4f1) in BMDM stimulated with CM compared to TGF-β1 in vitro (Fig. 4C and fig. S8E), showing that cancer condition is a critical factor for initiating MNT.

Unbiased bioinformatic analysis of the up-regulated DEGs of MNTs in Fig. 1B suggested gene network associated with neurotransmitter release (*37*) and receptor expression (Fig. 4D and fig. S9) (*38*). We found that expressions of presynaptic membrane calcium ion sensors (Syt13 and Syt14), neurotransmitter synthetases (Aldh9a1 and Dagla), and functional markers of sensory neuron (TrkA and Prph) are highly enriched in the MNTs as shown by the macrophage lineage scRNA-seq analysis (figs. S9 and S10A), implying a potential role of MNTs in the tumoral sensations that may contribute to cancer pain. Thus, we further characterized the biological function of MNTs according to their responses upon nociceptive stimulus in vitro (*39*, *40*) by imaging calcium efflux with OGB-1 (*31*). Intriguingly, we detected calcium flux of the BMDM-derived MNTs (Tubb3^+^ tdTomato^+^ cells) in response to the classic nociceptive stimulus capsaicin [an agonist of TRPV proteins that respond to cancer pain sensation (*41*)], whereas no response was detected in the unstimulated BMDM in vitro (Fig. 4, E and F; movie S2; and fig. S11). In addition, we found that the BMDM-derived MNTs also respond to the stimulations of GSK1016790A (agonist of nociceptor Trpv4) (*42*) and KCl (agonist of neuronal voltage-gated calcium channels) (*43*, *44*), supported by the up-regulation of Trvp4 and neuronal voltage-gated calcium channels (Cacna1a, Cacna1b, and Cacna1c) and activation of neuronal calcium signaling (p-CREB and Camk2d) (*45*–*47*) in both human and mouse MNTs (figs. S10, B to D, and S12).

Therefore, we investigated the potential functional role of MNTs by adoptively transferring in vitro generated BMDM-derived MNTs (Fig. 4B) into the nonobese diabetic/severe combined immunodeficient (NOD/SCID) mice with malfunctional background macrophage in a gain-of-function manner. As shown in Fig. 4G, local adoptive transfer of MNTs markedly increased the cancer-associated nociceptive response of LLC-bearing NOD/SCID mice according to their total spontaneous nocifensive behaviors in vivo. Consistently, we detected the activation of neuronal calcium signaling (p-CREB and Camk2d) (*45*–*47*) in both human and mouse MNTs (fig. S12). More interestingly, we detected a specific expression of pain receptors Ano1 (*48*) and Trpc1 (*49*) in the MNTs (Fig. 4H) and connection of synaptophysin-expressing MNTs in the LLC tumor in vivo and neurocircuit formation of BMDM-derived MNTs in vitro (fig. S13), evidencing the involvement of MNTs in the TME for cancer pain promotion. These data suggested that MNTs potentially exhibit nociceptor-like activities in tumors, which may represent a potential therapeutic target for cancer pain.

### Smad3 is essential for MNT development

**Fig. 5.**
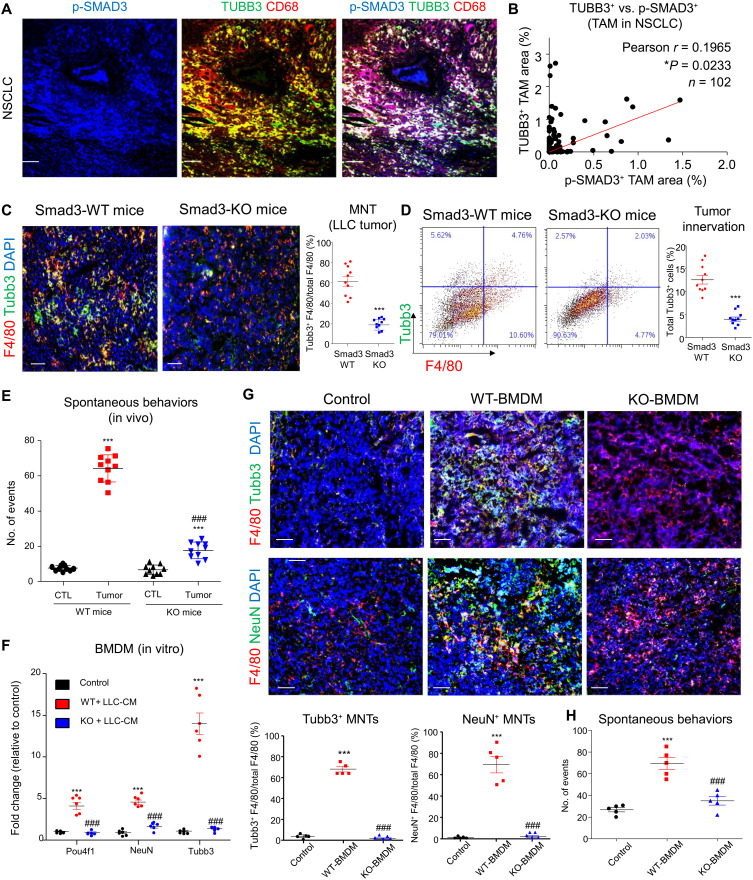
Smad3 is essential for MNT formation in the TME. (**A**) Representative image of the NSCLC cohort shows hyperactivation of SMAD3 (p-SMAD3, blue) in human TUBB3^+^CD68^+^ MNTs as shown by confocal imaging. (**B**) NSCLC cohort reveals a positive correlation between TAM-specific SMAD3 activation (p-SMAD3^+^CD68^+^) and MNT abundancy in NSCLC, detected by quantification of % stained area in multiplex immunohistochemistry (*P = 0.0233, Pearson correlation = 0.1965; *n* = 102). Reduction of mouse Tubb3^+^F4/80^+^ MNTs in the LLC tumor of mice with Smad3-KO TME (Smad3-KO mice), detected by (**C**) immunofluorescence and (**D**) flow cytometry analysis [*n* = 5 (duplicate); ***P < 0.001 versus Smad3-WT, *t* test]. (**E**) LLC cell inoculation markedly induces cancer-associated nocifensive behaviors (total counts of flinching and licking in 30 min) in the WT mice, which is significantly prevented in the Smad3-KO mice [***P < 0.001 versus tumor-free control (CTL); ^###^*P* < 0.001 versus tumor-bearing WT mice, one-way ANOVA; *n* = 5 (duplicate)]. (**F**) LLC-CM Smad3 dependently induces de novo mRNA expression of neuronal differentiation markers Pou4f1, NeuN, and Tubb3 in BMDMs in vitro (***P < 0.001 versus Control, ^###^*P* < 0.001 versus WT + LLC-CM, one-way ANOVA; *n* = 6). Local adoptive transfer of WT BMDMs (WT-BMDM) increases (**G**) neuronal marker expressing MNTs (F4/80^+^ Tubb3^+^, F4/80^+^ NeuN^+^) in the LLC tumors of NOD/SCID mice, (**H**) contributing to the significant increase of their cancer-associated nocifensive behaviors (total counts of flinching and licking observed in 30 min), which are effectively canceled in mice that received Smad3-KO BMDMs (KO-BMDM) (***P < 0.001 versus Control, ^###^*P* < 0.001 versus WT-BMDM, one-way ANOVA; *n* = 5). Scale bars, 50 μm.

Smad3 has been suggested to regulate neural lineage commitment of multipotent neural stem cells (*50*) and nociception in chronic pancreatitis (*28*). We also unexpectedly detected a strong association between the up-regulated genes of the in vitro TGF-β1/Smad3–activated BMDMs with neurogenesis by scRNA-seq in a kidney disease study (*24*). Here, we evidenced hyperactivation of SMAD3 in human MNTs of NSCLC biopsies by confocal microscopy (Fig. 5A), and the association between macrophage-specific Smad3 and the abundance of MNTs in the human NSCLC (*P = 0.023, *r* = 0.1965, *n* = 102) by Opal multiplex immunohistostaining (Fig. 5B). Therefore, we examined the regulatory role of Smad3-dependent TME in MNT in the experimental lung cancer models. We found that not only MNT but also the tumor-associated nocifensive behaviors were largely suppressed in the LLC tumor with Smad3-knockout TME (Smad3-KO mice) in vivo (Fig. 5, C to E), implying a crucial role of Smad3-dependent TME in the MNT regulation. We further tested the functional role of macrophage-specific Smad3 in MNT by adoptively transferring Smad3–wild-type (WT) or Smad3-KO BMDMs into macrophage-malfunctioned NOD/SCID mice (*23*). The LLC-CM–induced neuronal marker expression in the WT BMDMs was markedly prevented by Smad3 deletion in vitro (Fig. 5F). Furthermore, as shown in Fig. 5 (G and H), adoptive transfer of BMDM markedly increased MNT and cancer-associated nocifensive behaviors in the LLC-bearing NOD/SCID mice, which was markedly suppressed in the group that received BMDMs lacking Smad3 (KO-BMDM), suggesting the importance of Smad3 in MNT.

### Inhibition of Smad3 blocks MNT-mediated neurogenesis in experimental lung carcinoma

**Fig. 6.**
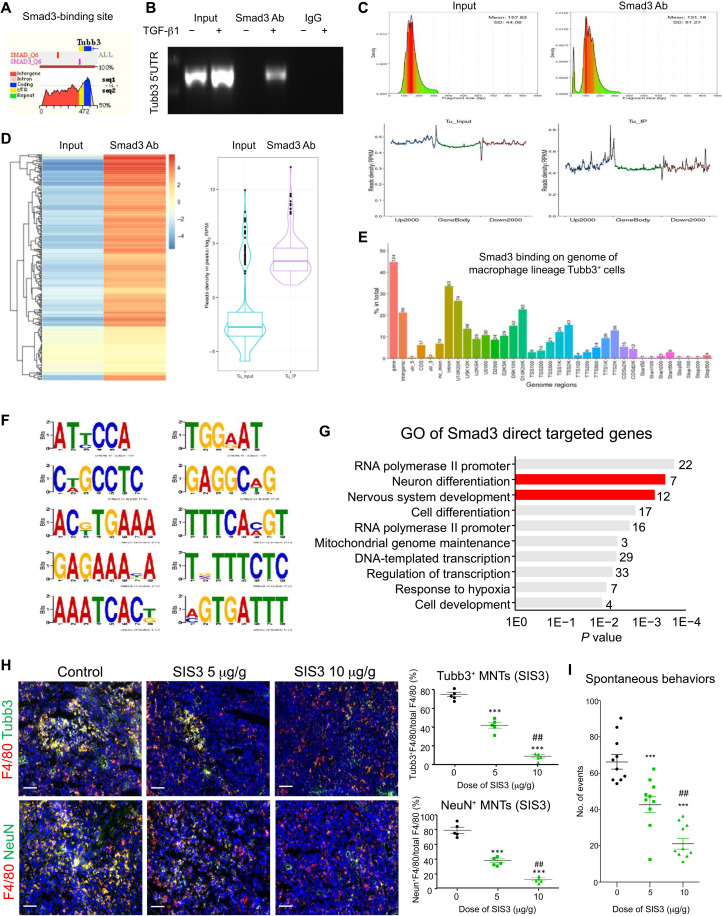
Targeting Smad3 effectively blocks MNT-driven neurogenesis in vivo. (**A**) ECR browser detects a direct binding site of Smad3 of the conserved region of mouse and human Tubb3 genomic sequence at 5′UTR (purple). (**B**) TGF-β1 (5 ng/ml) stimulation enriches the physical interaction of Smad3 protein on the 5′UTR of Tubb3 genomic sequence in BMDM in vitro as confirmed by ChIP-seq. The Tubb3^+^LysM-tdTomato^+^ MNTs are FACS-collected from the LLC-bearing LysM-Cre/ROSA-tdTomato mice, and their Smad3-bound chromatins are pulled down and submitted for DNA sequencing. The identified Smad3 binding sites in the genome of the in vivo MNTs are shown as (**C**) density distribution reads per kilobase million (RPKM) plot, (**D**) heatmap, (**E**) genome region chart, and (**F**) motif analysis. (**G**) There are 7 neuron differentiation and 12 nervous system development-related genes (red) detected as Smad3 direct target genes in the tumoral MNTs as revealed by GO analysis. Treatment with the pharmaceutical Smad3 inhibitor SIS3 dose-dependently blocks the formation of (**H**) Tubb3^+^ and NeuN^+^ MNTs and (**I**) cancer-associated spontaneous nocifensive behaviors (total counts of flinching and licking observed in 30 min, duplicate) of the LLC-bearing mice in vivo [***P < 0.001 versus solvent control, ^##^*P* < 0.01 versus SIS3 (5 μg/g), one-way ANOVA; *n* = 5]. Scale bars, 50 μm.

Neurogenesis is crucial for cancer pain development (*51*) and tumor progression (*52*), where tumoral Tubb3 expression and MNT levels (Fig. 2F) are highly associated with the mortality in NSCLC. Mechanistically, we identified that TGF-β1 triggered direct binding of Smad3 on the 5′ untranslated region (5′UTR) of Tubb3 genomic sequence in BMDM in vitro by ECR browser and chromatin immunoprecipitation–polymerase chain reaction (ChIP-PCR) assay (Fig. 6, A and B), suggesting a regulatory role of Smad3 in the TGF-β1–driven neurogenesis at the transcriptional level. Thus, we further elucidated the regulatory importance of Smad3 in MNT in a comprehensive manner by conducting in vivo ChIP-sequencing (ChIP-seq). In brief, the live macrophage lineage–derived Tubb3^+^ MNTs were FACS (fluorescence-activated cell sorting)–collected from the LLC tumor–bearing LysM-Cre/ROSA-tdTomato mice (fig. S5B); their Smad3-bound chromatin DNA fragments were pulled down and submitted for deep sequencing (Fig. 6, C to F, and fig. S14). The peaks of Smad3 binding were called by the Model-based Analysis of ChIP-Seq (MACS) program, normalized with input samples, and filtered by *P* value at the significance level >10^−13^, where 7 neuron differentiation and 12 nervous system development–related genes were identified out of the total 212 direct Smad3 targets in the MNTs (Fig. 6G and file S3), uncovering a previously unidentified pathogenic role of Smad3 in the MNT-mediated neurogenesis. We demonstrated that pharmaceutical inhibition of Smad3 with the specific inhibitor SIS3 effectively blocked MNT as well as the associated tumor innervation (Tubb3^+^ and NeuN^+^ cells) and cancer-associated spontaneous nocifensive behaviors of the LLC-bearing mice (Fig. 6, H and I). Thus, Smad3 may represent a druggable target for targeting MNT-driven neurogenesis in cancer.

## DISCUSSION

Cancer is still the top leading cause of death worldwide. Pain is one of the common complications that not only severely affects the life quality of patients physically and mentally but also markedly increases the mortality in a number of cancer types (*1*). However, treatments are ineffective because the pathogenic mechanism of tumor innervation is still largely unclear (*26*, *27*, *53*). Here, inspired by our scRNA-seq analysis of macrophages in vitro (*24*), we unexpectedly discovered a direct mechanism of TAM for promoting de novo neurogenesis in TME. Our findings revealed that TAM can directly transit into neuron-like cells via a previously unknown phenomenon MNT in NSCLC, especially older adenocarcinoma patients, which is in line with the Ayala *et al.* (*26*) study that neurogenesis of tumor peripheral zone increases with age. The MNTs showed calcium channel activities and neuron development under cancer conditions in vitro and ex vivo. Mechanistically, we identified a crucial role of Smad3 in MNT development. Consistently, we further demonstrated that both macrophage-specific and pharmaceutical inhibition of Smad3 effectively blocked MNT in vivo, which may serve as a druggable target for suppressing neurogenesis in lung carcinoma.

Increasing evidence suggests that numerous non-neuronal cell types play an important role in pain sensation, including cancer cells and macrophages (*4*). A recent study demonstrated that resident microglia and peripheral monocytes act synergistically to initiate hypersensitivity and promote chronic pain after peripheral nerve injury (*16*), where macrophage depletion largely suppressed mechanical and cold hypersensitivity, demonstrating the role of macrophage in evoked pain (*17*, *18*). However, direct mechanism of macrophage in the development and regulation of cancer pain is still largely unknown. Therefore, in addition to macrophage depletion by liposome clodronate, we demonstrated macrophage’s direct and causal role in cancer pain by local adoptive transfer of in vitro derived BMDM and MNTs into LLC tumor, significantly promoting the nocifensive behaviors of the LLC-bearing recipient mice. Using in vitro scRNA-seq, we uncovered a number of gene regulatory networks responsible for macrophage transition and activity (*24*). Unexpectedly, these TGF-β1–induced transcriptomes in BMDMs are highly associated with neuron differentiation, as suggested by an unbiased gene regulatory network analysis (*24*). The major regulatory network “Ephrin-B, PAK1, NCK2 (Grb4), EGFR, FISH” is highly associated with biological processes including axonogenesis, cell morphogenesis involved in neuron differentiation, axon development, neuron projection morphogenesis, and axon guidance, supported by the published experimental evidence in the MetaCore analytical suite database (*24*). In addition, these DEGs were unexpectedly associated with cancer by GO analysis. In this study, we found that TGF-β1 was able to trigger the expression of neuronal differentiation marker Tubb3 and neuron development transcription factor Pou4f1 in BMDMs (*54*, *55*), which was further promoted by the cancer cell–derived secretome in vitro. Tubb3 is essential for axon function and maintenance (*56*) and specifically expressed in neuron (*57*), serving as an ideal marker to identify neuron-like phenotypes of BMDM. The mature neuron morphology with dendrites and axon extension was only induced by the cancer cell–derived secretome in vitro (*26*, *27*, *58*), suggesting that additional factor from cancer cell–derived secretome is required for MNT, which is in contrast to macrophage-myofibroblast transition that can be solely induced by TGF-β alone (*24*, *25*).

To investigate TAMs in vivo, we used a well-established LysM promoter for TAM-specific Cre induction as other macrophage studies in cancer (*59*–*61*), bone development (*62*, *63*), macrophage differentiation (*64*), and atherosclerosis (*65*). We found that diphtheria toxin–mediated macrophage depletion substantially reduced Tubb3^+^ cells in the TME, which can be recovered by the adoptive transfer of BMDM in vivo. Fate-mapping study demonstrated the existence of TAM-derived neuronal marker–positive cells in the LLC tumors, where >90% of the Tubb3^+^ cells were derived from macrophage lineage, and Tubb3^+^ expression was observed in adoptively transferred GFP^+^ BMDMs. According to the well-documented epithelial-mesenchymal transition (*66*, *67*) and our experiences in macrophage transition (*24*, *25*, *55*, *68*), loss of parental cell markers will be found at the completion of cell type transition. In line with this notice, we detected that MNTs completely lost their macrophage markers (Cd68, F4/80, Cd74, Ccl9, Ccr5, Csf1r, H2-Eb1, and H2-Ab1) (*25*). The development of Tubb3^+^ cells from macrophage via intermediate MNTs with both phenotypes was recapitulated by several trajectory analyses of macrophage lineage scRNA-seq including pseudotime identifying cell fate decision by spotting switch-like change gene expression pattern (*69*) and the more advanced RNA velocity predicting the future state of individual cells with the model of transcription dynamics, showing cell differentiation in terms of spliced mRNA abundance (RNA velocity) (*33*), therefore identifying the previously unknown phenomenon MNT.

It is well documented that TAMs can influence pain sensation of the host via indirect mechanisms (*5*). For example, macrophages can affect analgesia by indirectly releasing anti-inflammatory mediators such as IL-10 and specialized pro-resolution mediators (*14*, *15*). The emerging role of macrophages in periphery pain regulation has recently been suggested, as macrophage-specific depletion impaired development of mechanical and thermal hypersensitivity and delayed resolution of inflammatory pain in vivo (*13*, *16*). However, the underlying mechanism of TAM in cancer-associated nociceptive pain is still largely unknown. This is the first study to investigate a potential direct mechanism of TAM for promoting nociception in cancer via de novo neurogenesis in the TME. Unexpectedly, using two-photon confocal live imaging, we demonstrated that MNTs have neuron-like morphology and phenotypes including calcium efflux and nociceptive activity under cancer condition ex vivo and in vitro. Unbiased gene network analysis revealed the neurotransmitter release function of MNTs; a synaptotagmin-centric functional subnetwork was reconstructed with the up-regulated DEGs of the MNTs in vivo. Synaptogenesis is essential for connecting neurons in neurocircuits via synapses, which is guided by neurotransmitter release (*70*). We found that not only synaptotagmins (Syt13 and Syt14) but also neurotransmitter synthetases (Dagla and Aldh9a1) and synapse markers (Syp, PSD95/Dlg4, and Map2) are highly expressed in the MNTs. In addition to classic nociceptor Trpv1, our GO analysis revealed pain mediator Trpv4 from 55 of the 279 up-regulated DEGs associated with ion homeostasis (*24*, *71*). As both Trpv1 and Trpv4 were expressed by MNTs, and responsive to pain inducer capsaicin (*41*, *72*, *73*), their roles in the MNT-mediated cancer pain are worth further elucidation for developing novel analgesics. We have taken the advantages of OGB-1 calcium imaging to examine the neuronal activities of MNTs in this study compared to the patch clamp–based electrophysiological experiments, including simultaneously monitoring signals of multiple cells, mapping calcium activity in subcellular compartments of neuron (cell body, axon, and dendrites), and intrinsic signal amplification by calcium-induced calcium release from intracellular store (*74*, *75*). We finally observed a connection of MNTs in the TME, which may be important for executing their functional roles via neurocircuit formation for promoting cancer pain. This study was mainly focused on the functional role and regulatory mechanism of MNTs with subcutaneous tumor models in a short experimental period, where injury to the central nervous system was not induced. Further elucidation should be continued to uncover the potential roles of MNT in the macrophage-dependent evoked pain and other human diseases.

Pain and inflammation are bidirectionally enhanced during disease progression (*11*). For example, nociceptive sensory neurons not only respond to immune signals but also directly modulate inflammation, evidenced by the silencing of nociceptors that reduce inflammation in the allergic airway (*11*). We recently identified the previously unknown phenomenon macrophage-myofibroblast transition, which also originated from the bone marrow (*29*, *76*), where tissue-infiltrating BMDMs can be further differentiated into fibrogenic myofibroblasts locally at inflammatory kidney (*24*, *77*) and TME (*25*) in a TGF-β1/Smad3–dependent manner. Macrophages are highly dynamic and exhibit various phenotypes depending on the microenvironment (*78*, *79*), including proinflammatory M1-like and anti-inflammatory M2-like phenotypes, which may play distinct roles in the induction and resolution of pain (*5*). Our latest study revealed that the neuronal transcription factor Pou4f1 is a Smad3 target gene in BMDM under TGF-β1 stimulation in vitro and TGF-β1/Smad3 signaling also regulates the nociceptive pain in chronic pancreatitis (*28*), implying a potential regulatory role of Smad3 in neuronal phenotype at the genomic level (*55*). By applying our experiences from tissue inflammation into cancer, we have identified a Smad3-dependent TME that is critical for promoting cancer progression (*25*, *80*, *81*). We found that mice lacking Smad3 and receiving systemic Smad3 inhibitor treatment are protected against tumor growth, invasion, metastasis, and death on syngeneic mouse models in vivo. However, the regulatory roles and mechanisms of Smad3 in TME are still largely unknown, especially cancer pain. Here, we identified that Smad3 is essential for promoting MNT in TAMs. Smad3 directly binds to a number of genes responsible for neurogenesis in TAMs undergoing MNT in vivo, uncovered by the ChIP-seq analysis of the MNTs sorted from LLC tumor in vivo. In line with our notion, we demonstrated that macrophage-specific inhibition of Smad3 effectively blocked MNT and the cancer-associated spontaneous nocifensive behaviors in mice.

Effective treatments for cancer pain are urgently needed, which seriously affect quality of life, especially in patients with advanced cancer (*2*, *3*). In our previous work, we observed a marked reduction of tumor-friendly elements including angiogenesis, matrix metalloproteinase (MMP) production, and regulatory T cells in the Smad3-null or Smad3-inhibited TME (*25*, *80*). In this study, we further examined whether Smad3 can serve as a druggable target for controlling MNT. Encouragingly, pharmaceutical inhibition of Smad3 markedly suppressed MNT and the overall neurogenesis in tumor. To note, systematic targeting of Smad3 with SIS3 may impair T cell anticancer immunity that largely limits its translational development (*82*). This potential barrier can be overcome by directly blocking the key pathogenic regulators in the Smad3 downstream signaling in a disease- and cell type–specific manner. We have identified a number of previously unknown Smad3 downstream long noncoding RNAs and demonstrated their therapeutic potential in experimental inflammatory disease models in vivo (*83*–*86*). In addition, trace amount of MNT was observed in Smad3-KO TME, suggesting the potential development of MNT in prolonged Smad3 pharmaceutical inhibition; thus, the identification of Smad3-independent driver of MNT may help to better control MNT. Therefore, a better understanding of the underlying mechanism of MNT would uncover druggable targets for managing cancer pain.

Together, we identified MNT as a previously unknown and common phenomenon in the TME for promoting neurogenesis. The MNTs showed nociceptive activities under cancer condition ex vivo and in vitro. We finally identified the transcription factor Smad3 as a key regulator for initiating MNT in TAMs via transcriptional regulation at the genomic level. In concordance, we finally demonstrated that genetic and pharmaceutic targeting of Smad3 effectively blocks MNT-driven tumor innervation in mice. We believe that the discovery of MNT will provide important rationale for developing new clinical strategies to manage cancer pain.

## METHODS

### Experimental animals

All experimental procedures have been approved by the Animal Ethics Experimental Committee at the Chinese University of Hong Kong (AEEC no. 19/029/GRF) and conducted in accordance with the guidelines of AEEC and local regulations. Smad3-deficient mice (*Smad3^−/−^*) (*82*), macrophage-specific tdTomato overexpression transgenic mice, and NOD-SCID mice were used in this study.

### Generation of macrophage-specific tdTomato overexpression mice

C57BL/6 background LysM-Cre mice and tdTomato Cre-reporter transgenic mice (ROSA26-tdTomato) were purchased from The Jackson Laboratory. LysM-Cre/ROSA26-tdTomato mice were generated by crossing LysM-Cre and ROSA26-tdTomato mice. C57BL/6 background Smad3^−/−^ (exon 8 deletion and exon 7 disruption) and Smad3^+/+^ mice were provided by C. Deng (*82*). LLC syngeneic mouse tumor model was induced by subcutaneous inoculation of 2 × 10^6^ LLC cells into the right flank of a mouse. Mice were housed in a controlled environment (12-hour light/dark cycle, 23° ± 1°C, 60 to 70% humidity).

### Macrophage lineage-specific cell ablation

Diphtheria toxin (150 ng per mouse) (Sigma-Aldrich, D0564) was intraperitoneally injected to conditionally deplete macrophage in LysM-Cre Rosa26-iDTR mice as previously described (*87*). Clodronate and control liposomes (FormuMax Scientific Inc., F70101C-N) were intraperitoneally injected to C57BL/6 mice 2 days before tumor inoculation (initial dose: 200 μl per mouse) and repeated every 5 days (maintenance dose: 100 μl per mouse) until tumor collection (*88*).

### Spontaneous nocifensive behaviors observation

Observation began after more than 2 hours of acclimatization at transparent cages. In a 30-min observation, nocifensive behaviors including (i) flinching (the rippling or rotation of dorsal area as 1 count) and (ii) licking (the touching of the mouth and nose to the tumor area) were recorded (*32*, *89*); these behaviors are indicative of cutaneous discomfort or pain (*90*, *91*). Observation was conducted 1 day before scarification.

### Adoptive transfer studies

To determine the TAM-specific role of Smad3 in MNT-mediated tumor progression, phosphate-buffered saline (PBS), *Smad3^+/+^*, and *Smad3^−/−^* BMDMs were mixed with LLC cells in a 1:1 ratio (2 × 10^6^ cells per mouse) to induce local tumor via subcutaneous injection for three groups: control, WT-BMDMs, and KO-BMDMs. To determine the MNT-specific role in nociception, PBS- and BMDM-derived MNT cells [BMDM stimulated with TGF-β1 (5 ng/ml) for 5 days and 10% (v/v) LLC-CM for additional 7 days] in vitro were mixed with LLC cells in a 1:1 ratio (2 × 10^6^ cells per mouse) to induce local subcutaneous tumor for control and MNT groups.

### SIS3 treatment

LLC tumor (subcutaneous)–bearing Smad3^+/+^ mice were randomly divided into three groups: control, 5 μg/g SIS3, and 10 μg/g SIS3. SIS3 (S0447, Sigma-Aldrich) was administered daily via intraperitoneal injection until tumor collection, whereas the control group received solvent control (0.05% dimethyl sulfoxide) instead.

### Antibodies

Antibodies used for immunofluorescent staining, flow cytometry, and opal multiplexing are listed in table S1.

### TSA multiplexing and clinical samples

Formalin-fixed paraffin-embedded tissues and tumor microarray sections (5 μm thickness) were deparaffinized in xylene and rehydrated in descending alcohol gradient, followed by endogenous horseradish peroxidase (HRP) blocking in 3% (v/v in PBS) hydrogen peroxide solution (30 min) and heat-induced epitope retrieval in citrate buffer (95°C, 5 min, 0.0874 M sodium citrate dihydrate, 0.0126 M citric acid, pH 6.0). After incubation with CD68, TUBB3, and p-SMAD3 antibody overnight (4°C), sections were incubated in polymer-HRP–conjugated secondary antibody (Bio SB, BSB0005H) for 2 hours at room temperature, followed by Tyramide Signal Amplification (TSA)–488/546/647 (Thermo Fisher Scientific, B40953, B40954, and B40958) development. TSA-stained sections were captured on the Mantra quantitative pathology workstation (PerkinElmer, MA). Expression level and colocalization of the target proteins in terms of % area in captured images were quantified by inForm image analysis software (PerkinElmer, MA) with reference to unstained controls.

### NSCLC cohort

The formalin-fixed paraffin-embedded blocks of primary NSCLC samples from Prince of Wales Hospital, The Chinese University of Hong Kong (CUHK), were used for the study under the approved protocols (reference no. 2019.368) by CUHK Clinical Research Ethics Committee, and written informed consent was obtained from patients.

### Immunofluorescence

Optimal cutting temperature (OCT) compound (Sakura, Ref-4583)–embedded tumor tissues (5 μm thickness) were used for immunohistochemical staining. After OCT removal and blocking, sections were incubated with primary antibody overnight at 4°C. For nonconjugated primary antibody, sections were incubated with Alexa Fluor 488/546–conjugated secondary antibody (Thermo Fisher Scientific, 1:1000; α-Ms: F-2761, A-11003, α-Rb: A-11008, A-11010) in staining buffer for 2 hours at room temperature (eBioscience, 00-4222-26). Nuclei were stained with Hoechst 33342 (Thermo Fisher Scientific, H1399) and then mounted with PermaFluor medium (Thermo Fisher Scientific, TA-030-FM). Images were captured with a Zeiss fluorescence microscope and analyzed with ZEN image analysis software.

### Flow cytometric analysis

Tissues isolated from tumor-bearing mice were mechanistically dissociated, digested by Liberase (5401119001, Roche), filtered by 40-μm nylon mesh, and fixed with IC Fixation Buffer (00-8222-49, eBioscience) according to the manufacturer’s protocol to prepare single-cell suspension. MNT populations were stained with CD68-allophycocyanin (APC) (Santa Cruz Biotechnology) and TUBB3–fluorescein isothiocyanate (FITC) (Santa Cruz Biotechnology) antibodies overnight at 4°C. Flow cytometric data were acquired on LSRFortessa (Becton Dickinson) and analyzed in the Cytobank platform (cytobank.org) for quantitative analysis.

### Cell culture

The Lewis lung carcinoma (LLC, CRL-1642, American Type Culture Collection) cells with stable luciferase expression (LLC-luc) were cultured in Dulbecco’s modified Eagle’s medium (DMEM)/F12 (Invitrogen, 11320082) with 10% heat-inactivated fetal calf serum (FCS; PMC2044, Gibco), penicillin G (100 U/ml), and streptomycin (100 mg/ml; 15140122, Gibco). LLC conditioned medium (LLC-CM) was collected by overnight incubation of LLC cells (70 to 80% confluence) with serum-free DMEM/F12, followed by filtration with 0.2-μm nylon membrane. The neuronal model PC-12 cells were cultured in RPMI 1640 medium supplemented with 10% horse serum, 5% fetal bovine serum (FBS), and penicillin-streptomycin (100 U/ml) (*92*).

BMDMs were prepared following our well-established protocol (*77*, *93*). In brief, single-cell suspension of bone marrow cells was prepared from tibia, femur, and ilium bone. The bone marrow cells were differentiated for 7 days in DMEM/F12 with 10% heat-inactivated FCS and recombinant mouse macrophage colony-stimulating factor (M-CSF) (50 ng/ml; Gibco, PMC2044). The purity of BMDM culture was validated by flow cytometry as our first scRNA-seq study (*24*), where >95% of the cells expressed F4/80. MNTs were induced by further stimulating BMDM with TGF-β1 (5 ng/ml) for 5 days following 10% (v/v) LLC-CM incubation for 7 days. Mouse primary neuron was prepared according to previously established protocol (*92*). In brief, cortices were dissected and dissociated by 0.25% trypsin at 37°C for 20 min. Single-cell suspensions of primary neurons were prepared in neurobasal medium (Gibco) with 10% FBS and penicillin-streptomycin (100 U/ml) and allowed 24 hours for its attachment. Primary neurons can be used after 4 days of culture in neurobasal medium with 2% B-27 (Gibco) and 2 mM l-glutamine (Gibco).

### Quantitative real-time PCR

TRI reagent (TR 118, Molecular Research Center), reverse transcription system (A3500, Promega), and SYBR Green Supermix (#1725124, Bio-Rad) were used for RNA extraction, complementary DNA (cDNA) synthesis, and quantitative PCR, respectively, according to the manufacturer’s protocol. Pou4f1, NeuN, Tubb3, and Gapdh primers used in this study were listed in table S2. Gene expression levels normalized with glyceraldehyde-3-phosphate dehydrogenase (GAPDH) of three experiments were expressed as means ± SEM.

### Western blotting

The tissues or cells were lysed by RIPA Lysis Buffer System (Santa Cruz Biotechnology, sc-24948A). The protein concentration was measured by DC protein assay (Bio-Rad, #5000114), and 40 μg of protein was electrophoresed in 10% SDS-polyacrylamide gel (Bio-Rad, #1610149, #1610798, #1610799, and #1610801) and blotted to nitrocellulose membrane (Pall Corporation). The membrane was incubated with Pou4f1, NeuN, Tubb3, and GAPDH antibodies overnight 4°C. The bound antibody was detected by DyLight 800–conjugated secondary antibody (Rockland, α-Ms: 610-432-020, α-Rb: 611-132-122) with Odyssey infrared imaging system (LI-COR). The expression level of the target protein was quantified by ImageJ of three repeated experiments and normalized to the internal control (GAPDH).

### 10× scRNA-seq and transcriptomic analyses

TdTomato^+^ cells were FACS-sorted from single-cell digestion pooled from eight LLC tumors inoculated in four LysM/tdTomato mice and submitted for cell encapsulation and library construction by Chromium controller with 5′ expression kit (10x Genomics). The “Single-cell” sort mode was applied to exclude doublets in the single-cell suspension. Only cells with linear relationships in FSC-A and FSC-H plot were included in the sorting gate as fig. S5. The library was sequenced by Illumina NovaSeq 6000 platform (PE151bp, 660M raw read). The sequence data were converted into cloupe format by Cell Ranger v3.0.2, and DEGs of MNTs were generated by Loupe Cell Browser software. Leiden clustering was performed by scanpy. Developmental trajectories were reconstructed by Monocle 2 and MuTrans packages. Up-regulated DEGs of MNTs were submitted to Database for Annotation, Visualization, and Integrated Discovery bioinformatics resources (DAVID v6.8) for GO biological process enrichment analysis. The publicly available human NSCLC (fresh surgical resection of a squamous NSCLC tumor with live-cell selection by FACS) and mouse brain (cells isolated from a combined cortex, hippocampus, and subventricular zone) datasets and complete description of sample collection, library preparation, and sequencing configuration can be accessed in 10x Genomics website via the following URL: www.10xgenomics.com/resources/datasets/nsclc-tumor-1-standard-5-0-0 and https://support.10xgenomics.com/single-cell-gene-expression/datasets/3.0.0/neuron_10k_v3. Clustering of *t*-distributed stochastic neighbor embedding (*t*-SNE) was performed by Cell Ranger pipeline with default parameters based on the Van der Maaten method (*94*). Heatmaps were generated in Loupe Cell Browser by comparing single- and double-positive cells (CD68 and TUBB3) with count >1 in local mode.

### RNA velocity analysis

We used scVelo to infer future states of individual cells using the spliced and unspliced information. The aligned bam file generated by Cell Ranger was recounted with the Velocyto counting pipeline velocyto.py in python. The sample-wise counts of unspliced and spliced reads in loom format were loaded to scVelo. Genes with less than 20 spliced and unspliced counts in a cell were filtered, and the counts were normalized using normalize_per_cell(). A total of 2000 high variability genes were identified and retained by filter_genes_dispersion(), following which the counts were log-transformed using log1p(). The first- and second-order moments for each cell across its nearest neighbors were calculated using scvelo.pp.moments(). Subsequently, the velocities were estimated using the scvelo.tl.velocity() and velocity graph constructed using scvelo.tl.velocity_graph() function. Velocities were visualized on top of the diffusion UMAP space. The confidence values of the RNA velocity were computed with scVelo.tl.velocity_confidence() function. Scrublet was used to perform doublets analysis of the scRNA-seq dataset; doublet score higher than 0.4 was considered as doublet (*95*). The codes adopted for this paper are included as notes S1 and S2.

### ChIP assay

Bone marrow cells extracted from Smad3^+/+^ mice were treated with TGF-β1 (5 ng/ml) for 2 hours and then processed using the SimpleChIP Enzymatic Chromatin IP Kit (Cell Signaling Technology, #9003S) according to the manufacturer’s instruction. Antibodies against Smad3 and immunoglobulin G isotype control (Cell Signaling Technology) were used for immunoprecipitation. Specific primers used to detect Smad3 binding on mouse *Tubb3* gene in precipitated DNAs were included in table S2.

### ChIP sequencing

Tubb3^+^ tdTomato^+^ cells were FACS-sorted from the single-cell digestion of subcutaneous LLC tumor inoculated in LysM/tdTomato mice and then processed using the SimpleChIP Enzymatic Chromatin IP Kit and Smad3 antibody (Cell Signaling Technology, #9003S) according to the manufacturer’s instruction. Precipitated DNA concentration was measured by Qubit, and the fragment size was analyzed by agarose gel electrophoresis. DNA libraries were then paired-end sequenced by Illumina NovaSeq 6000.

### Two-photon microscopy

A custom-modified two-photon microscope (Scientifica, Uckfield, UK) was used for the ex vivo LLC tumor imaging with a Ti:sapphire femtosecond laser (Mai Tai DeepSee, Spectra-Physics, US), galvo-resonant scanners, and a Nikon ×16/0.80 numerical aperture water-immersion objective. With 930-nm excitation wavelength, the emitted fluorescent signals from OGB-1 (Invitrogen, O6807) and tdTomato were detected by photomultiplier tubes through a 520/20-nm band-pass filter and 609/34-nm band-pass filter, respectively.

### OGB-1 imaging in vitro

Bone marrow cells extracted from TdTomato^+^ mice were treated with M-CSF (50 ng/ml; Gibco) for 7 days to generate BMDMs and further stimulated with TGF-β1 (5 ng/ml; Gibco) for 5 days and LLC-CM for additional 7 days to induce MNTs. The in vitro OGB-1 image stacks were then acquired with the 1 μM capsaicin, 2 μM GSK1016790A, and 20 mM KCl stimulation of BMDMs and MNTs preloaded with 2 μM OGB-1. Fluorescent signals were recorded under an inverted microscope (Carl Zeiss Axio Observer) and analyzed with ZEN lite image analysis software and ImageJ (fig. S15). The calcium level [Δ*F*_*t*-0_(_Intensity difference between given time and 0 s_)/*F*_0_(_Intensity at 0 s_) of OGB-1 intensity] of three or more OGB-1^+^ cells in the field was calculated to show the change of calcium influx and visualized with ratiometric pseudocolor in representative images and video (*43*).

### Statistical analysis

Student’s *t* test or analysis of variance (ANOVA) with Bonferroni post-test was used to perform statistical analysis of the differences in Prism program (Prism 5.0, GraphPad Software, San Diego, CA). Pearson and Spearman correlation analysis and comparison of survival curves with log-rank test were used to analyze NSCLC cohort data. *P* < 0.05 was considered statistically significant.

## Supplementary Materials

### This PDF file includes:

Figs. S1 to S15 Notes S1 and S2 Tables S1 to S3

## Notes

View/request a protocol for this paper from *Bio-protocol*.
