# Supplementary material for: Single-cell RNA sequencing uncovers a neuron-like macrophage subset associated with cancer pain
Source: Sci Adv. 2022 Oct 7;8(40):eabn5535. doi: 10.1126/sciadv.abn5535 (PMC9544324; doi:10.1126/sciadv.abn5535)
Supplement: Supplementary file 1 — Figs. S1 to S15 Notes S1 and S2 Tables S1 to S3 [file sciadv.abn5535_sm.pdf]

Science Advances
AAAS

Supplementary Materials for

Single-cell RNA sequencing uncovers a neuron-like macrophage subset associated with cancer pain

Philip Chiu-Tsun Tang *et al.*

Corresponding author: Patrick Ming-Kuen Tang, patrick.tang@cuhk.edu.hk

Sci. Adv. **8**, eabn5535 (2022)
DOI: 10.1126/sciadv.abn5535

The PDF file includes:

Figs. S1 to S15
Notes S1 and S2
Tables S1 to S3
Legends for files S1 to S3
Legends for movies S1 and S2

Other Supplementary Material for this manuscript includes the following:

Files S1 to S3
Movies S1 and S2

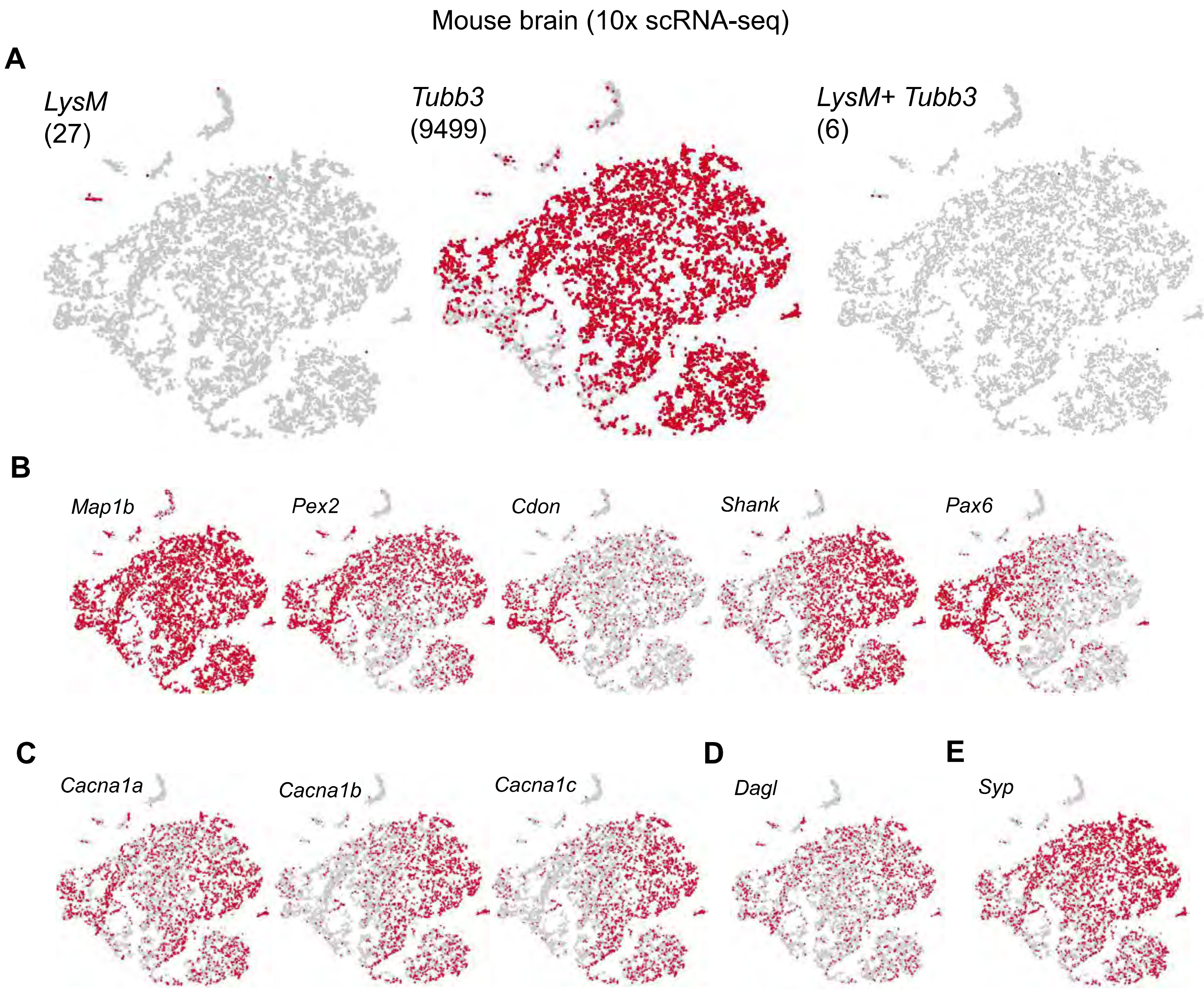

Fig. S1. Expression of LysM and neuronal genes in mouse brain.

(A) Expression of LysM was extremely low in the Tubb3^{+} cells in a mouse brain 10X scRNA-seq dataset (i.e. 6 out of 9499 = 0.063% of the total Tubb3^{+} cells), number of +ve cells were shown in bracket. **(B)** Neuron markers (Map1b, Pex2, Cdon, Shank, Pax6), **(C)** voltage gated calcium channel (Cacna1a, Cacna1b, Cacna1c), **(D)** neurotransmitter synthase (Dagla+Daglb) and **(E)** synapse marker (Syp) were highly expressed by Tubb3^{+} neurons in mouse brain.

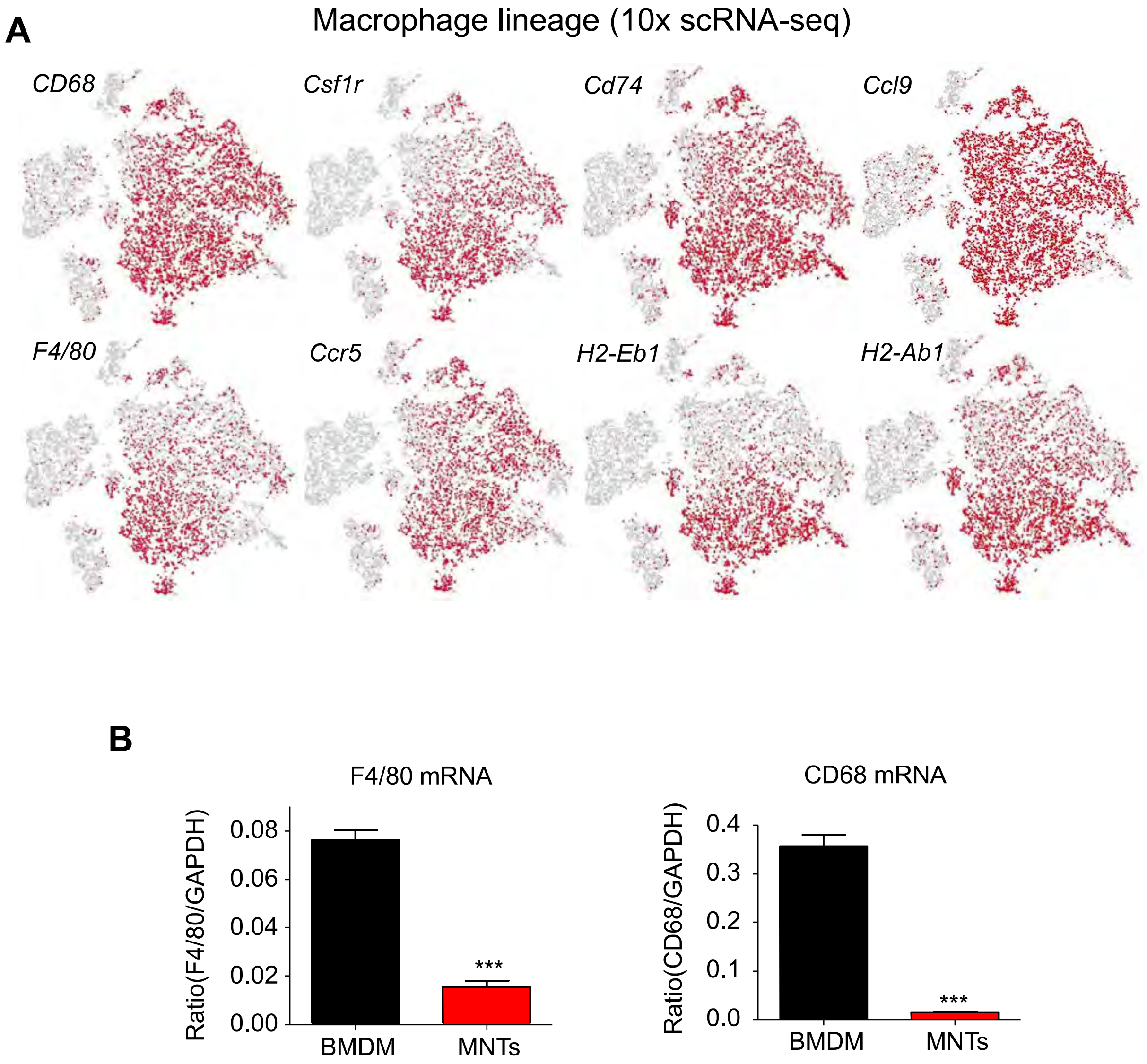

Fig. S2. **Expression levels of macrophage markers in MNTs.**

The expression levels of macrophage markers are low in the **(A)** MNTs of LLC-tumor *in vivo* (cluster #4 of Fig. 1A) and **(B)** BMDM-derived MNTs *in vitro*, detecting by 10X scRNA-seq and real-time PCR (n=4, ***$p<0.001$ vs BMDM, t-test) respectively.

A

NSCLC (10x scRNA-seq)

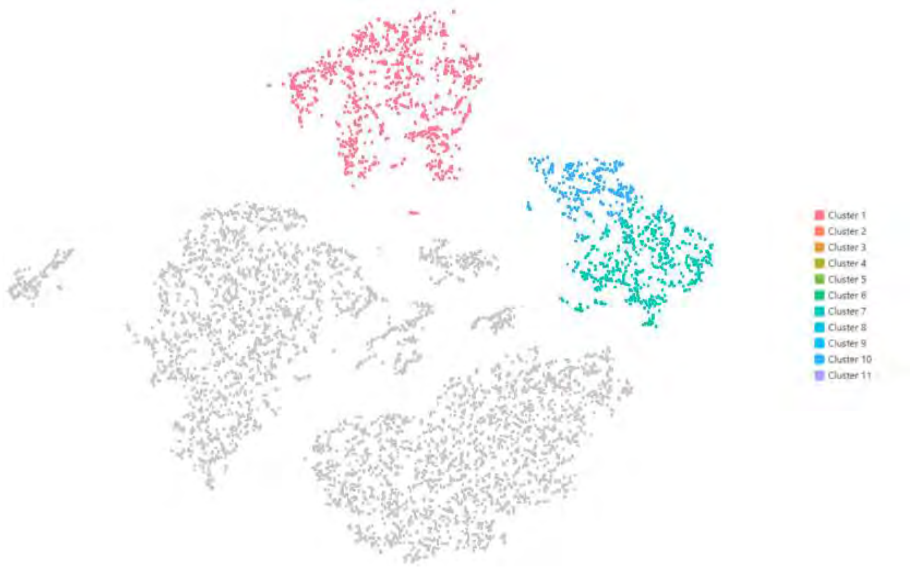

B

Cluster 1<br>TUBB3+	Cluster 10<br>CD68+	Cluster 7<br>TUBB3+ CD68
KRT7	*VCAN*	*C1QB*
KRT17	*PLIN2*	*C1QA*
PTGES	*FCN1*	*C1QC*
TRIM29	*SLC11A1*	*CLEC10A*
S100A2	*MCEMP1*	*MS4A6A*
FXYD3	*CLEC4E*	*CSF1R*
S100A16	*TREM1*	*MS4A4A*
KRT18	*TIMP1*	*S100B*
PCSK1	*S100A12*	*FCER1A*
S100A14	*THBS1*	*CPVL*

Fig. S3. scRNA-seq reveals MNT in NSCLC.

Top 10 specific marker genes (**B**) were extracted from MNT related clusters (**A**, Cluster 1, 7, 10) in NSCLC.

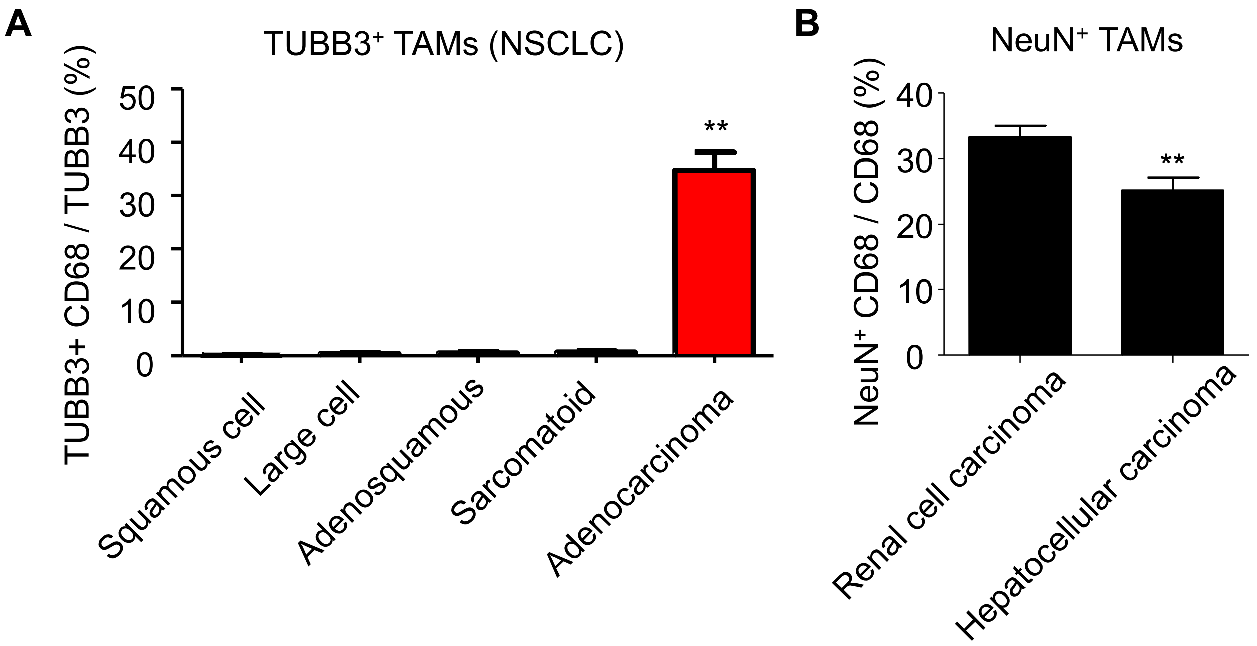

Fig. S4. (**A**) Distribution of TUBB3+ TAMs in NSCLC patients (n=102, **p<0.01 vs other NSCLC subtypes, one-way ANOVA). (**B**) Quantification of NeuN+ TAMs in renal cell carcinoma and hepatocellular carcinoma patient biopsies of Fig. 2B (n=30, **p<0.01 vs renal cell carcinoma, t-test).

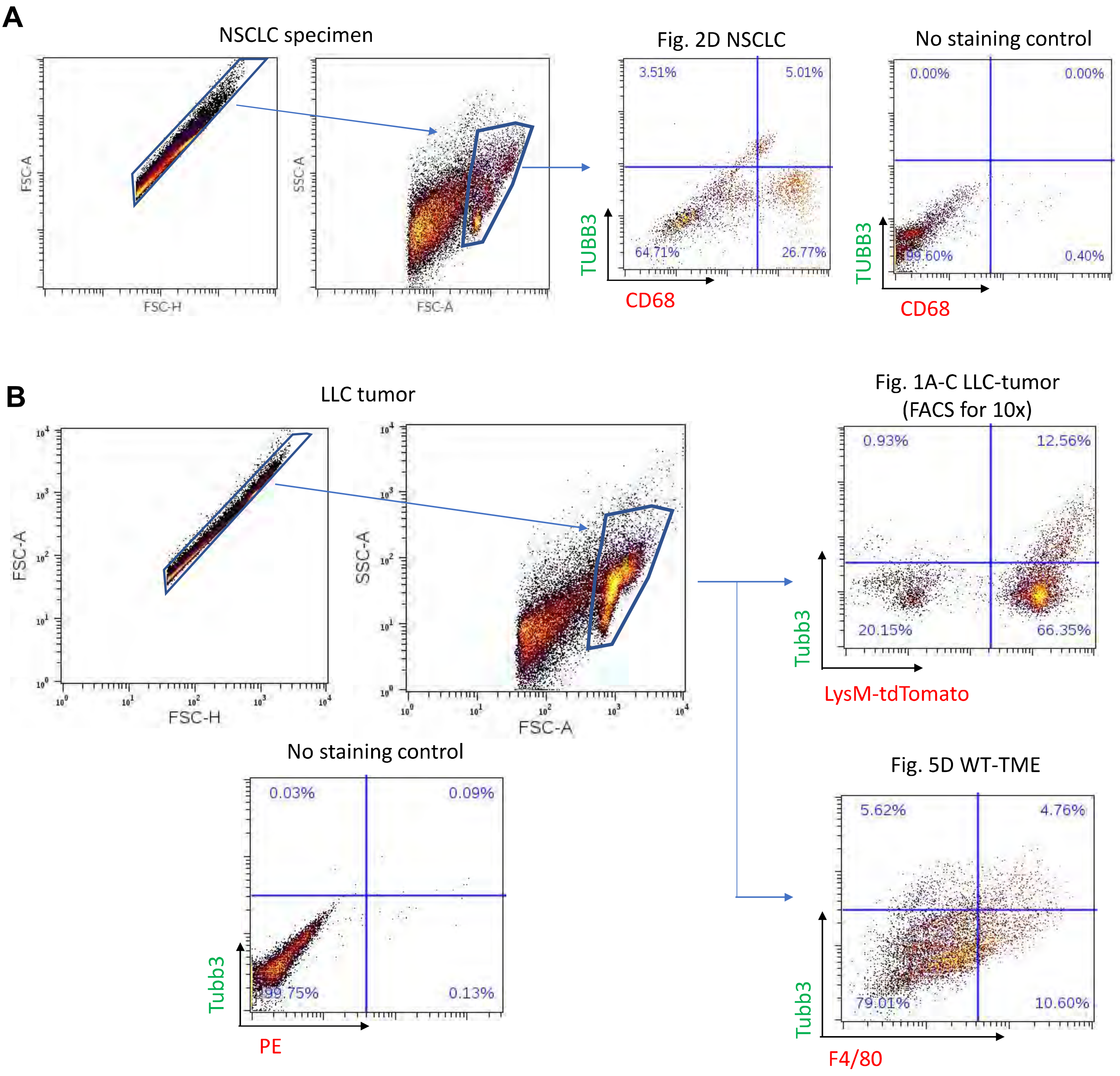

Fig. S5. Gating strategy for flow cytometric analysis of MNTs in (**A**) human NSCLC and (**B**) mouse LLC tumor.

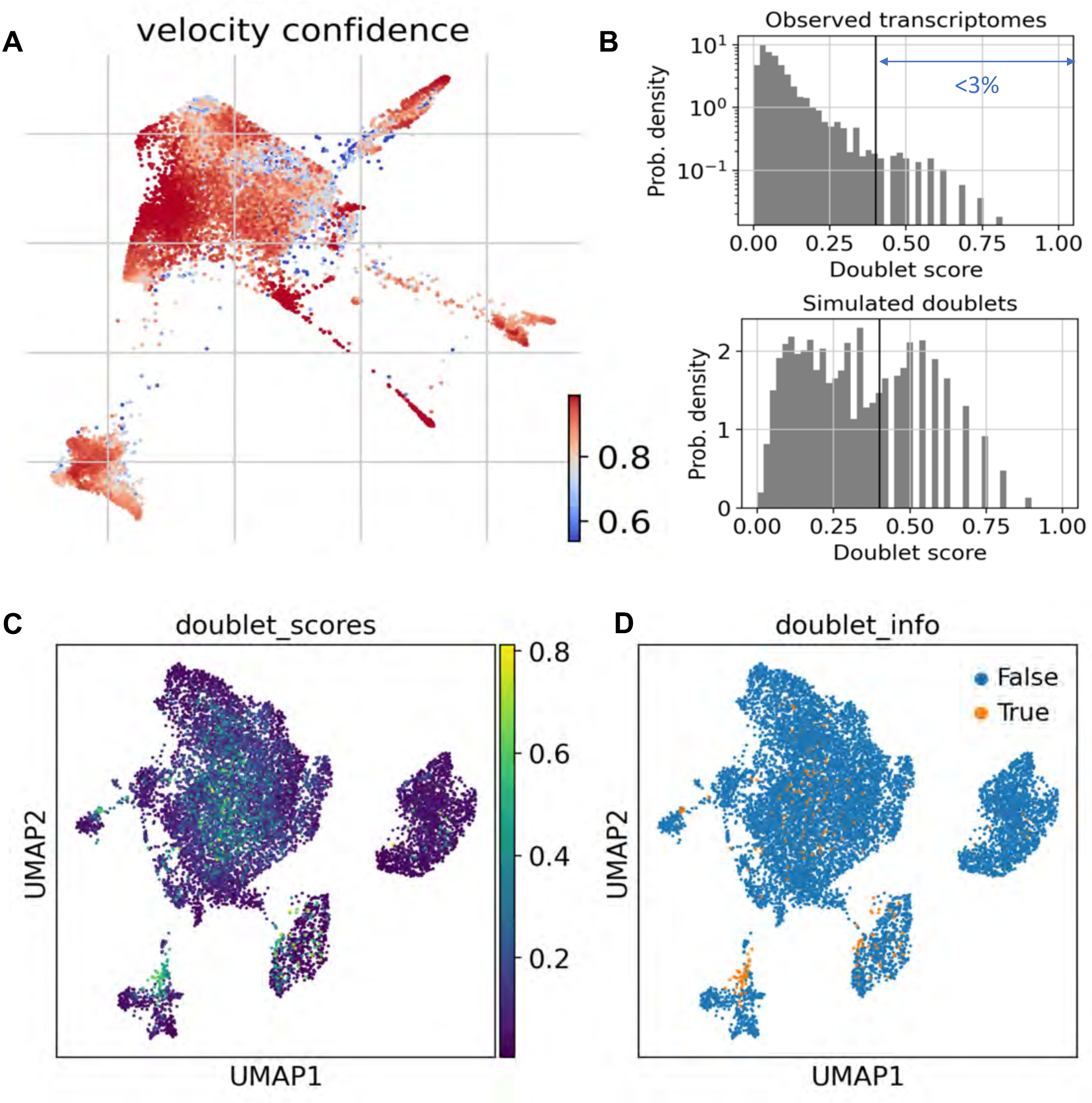

Fig. S6. Quality control of the macrophage lineage scRNA-seq.

(A) Most of the macrophage-lineage cells in RNA velocity analysis (Fig. 3A) showing velocities with high confidence values (>0.8). In Scrublet doublet analysis, (**B**) >97% of cells with doublet score <0.4, (**C**) particularly the MNT derived cluster 4, **(D)** indicating it is not a doublet-derived cluster (blue cluster).

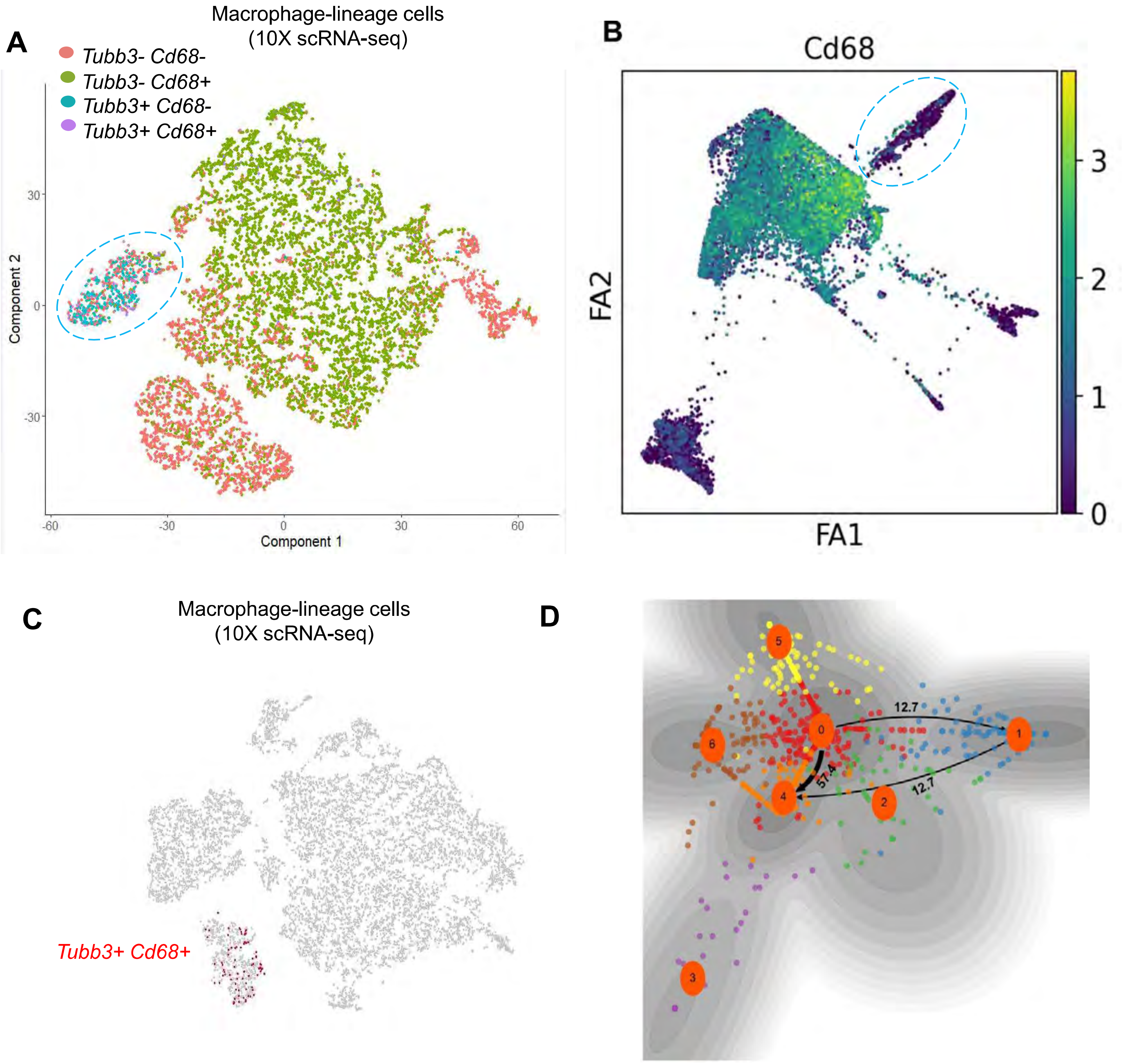

Fig. S7. Trajectory analyses evidences the MNT.

The Tubb3^{+} Cd68^{+} cells were commonly found in the t-SNE plots of (**A**) purple high-lighted in Fig. 3D, (**B**) cluster 4 in Fig. 3A, and (**C**) red high-lighted in Fig. 1B by analyzing the same 10X scRNA-seq dataset of the FACS-isolated macrophage-lineage cells from Day 15 LLC-tumor with various bioinformatic strategies. (**D**) A diffusion map further confirmed the result of MNT (Cluster 0 to 4) shown in the Fig. 3A by MuTrans analysis.

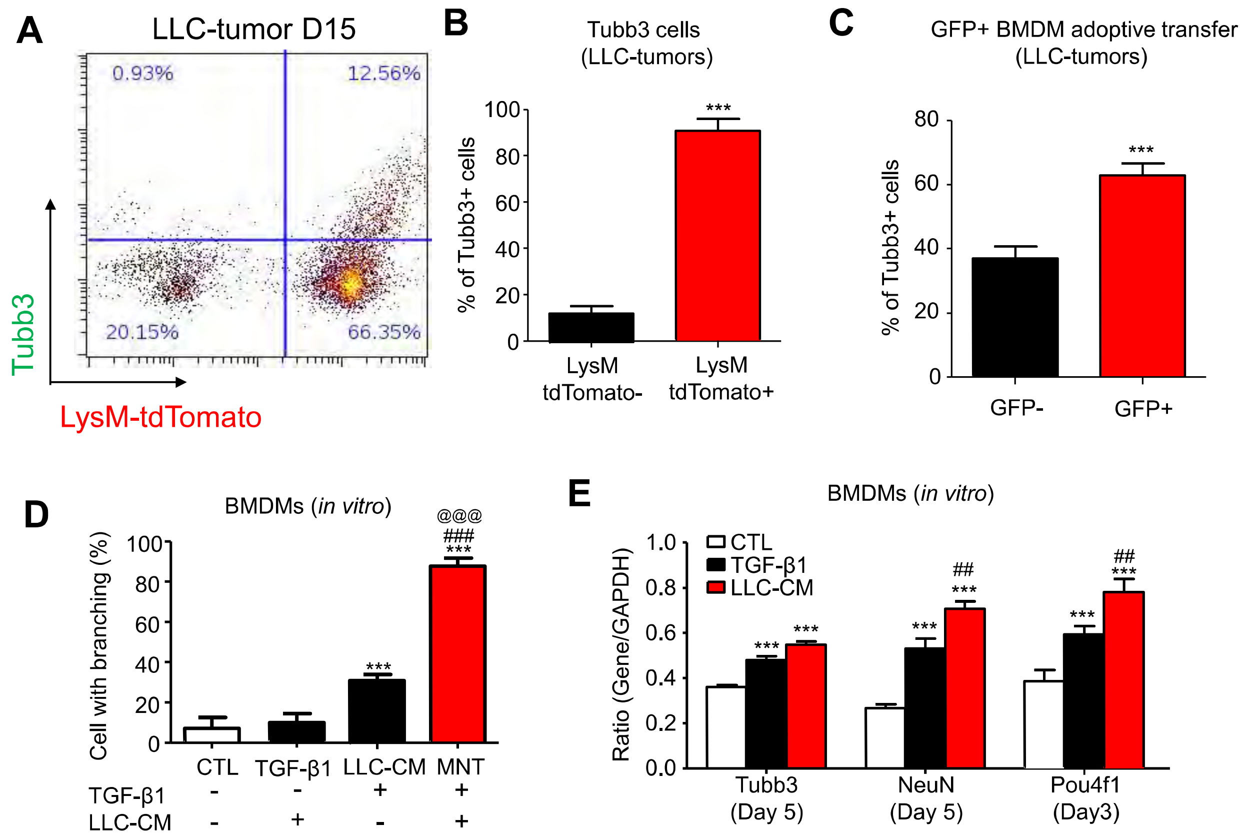

Fig. S8. Quantification of macrophage derived MNTs.

(**A-B**) The macrophage-lineage derived Tubb3$^+$ cells in Fig. 3E is quantified by flow cytometric analysis (n=4, ***p< 0.001 vs LysM-tdTomato -ve, t-test). **(C)** Quantification of GFP$^+$ BMDM-derived Tubb3$^+$ cells in LLC-tumor of Fig. 3F (n=5, ***p<0.001 vs GFP -ve, t-test). Effects of TGF-β1 and LLC-CM on the MNT development of BMDM *in vitro*, showing by the induction of **(D)** neuron-like branching in Fig. 4B and **(E)** neuron markers expression in Fig. 4C (n=4, ***p<0.001 vs CTL, ##p<0.01 ###p<0.001 vs TGF-β1, @@@p<0.001 vs LLC-CM, one way ANOVA).

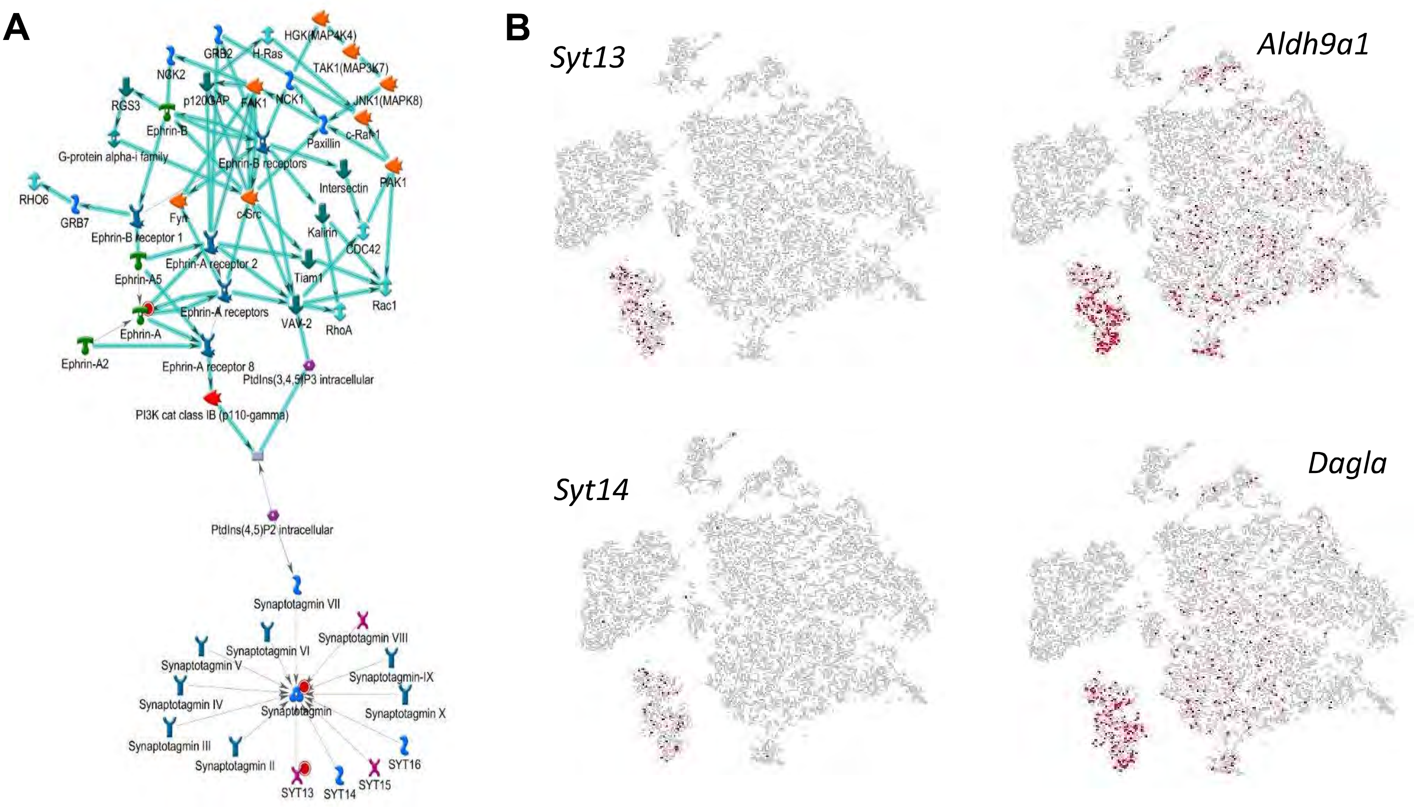

Fig. S9. Expression of neuronal functional marker genes in MNTs.

(**A**) A regulatory gene network centric with synaptotagmins, a calcium dependent regulator of neurotransmitter release, was unbiasedly reconstructed with the upregulated DEGs of MNTs in Fig 1B by MetaCore bioinformatic platform. (**B**) Consistently, synaptotagmins (Syt13, Syt14) and neurotransmitter synthases (Aldh9a1, Dagla) are highly expressed in the MNTs *in vivo* showing by macrophage lineage specific scRNA-seq analysis.

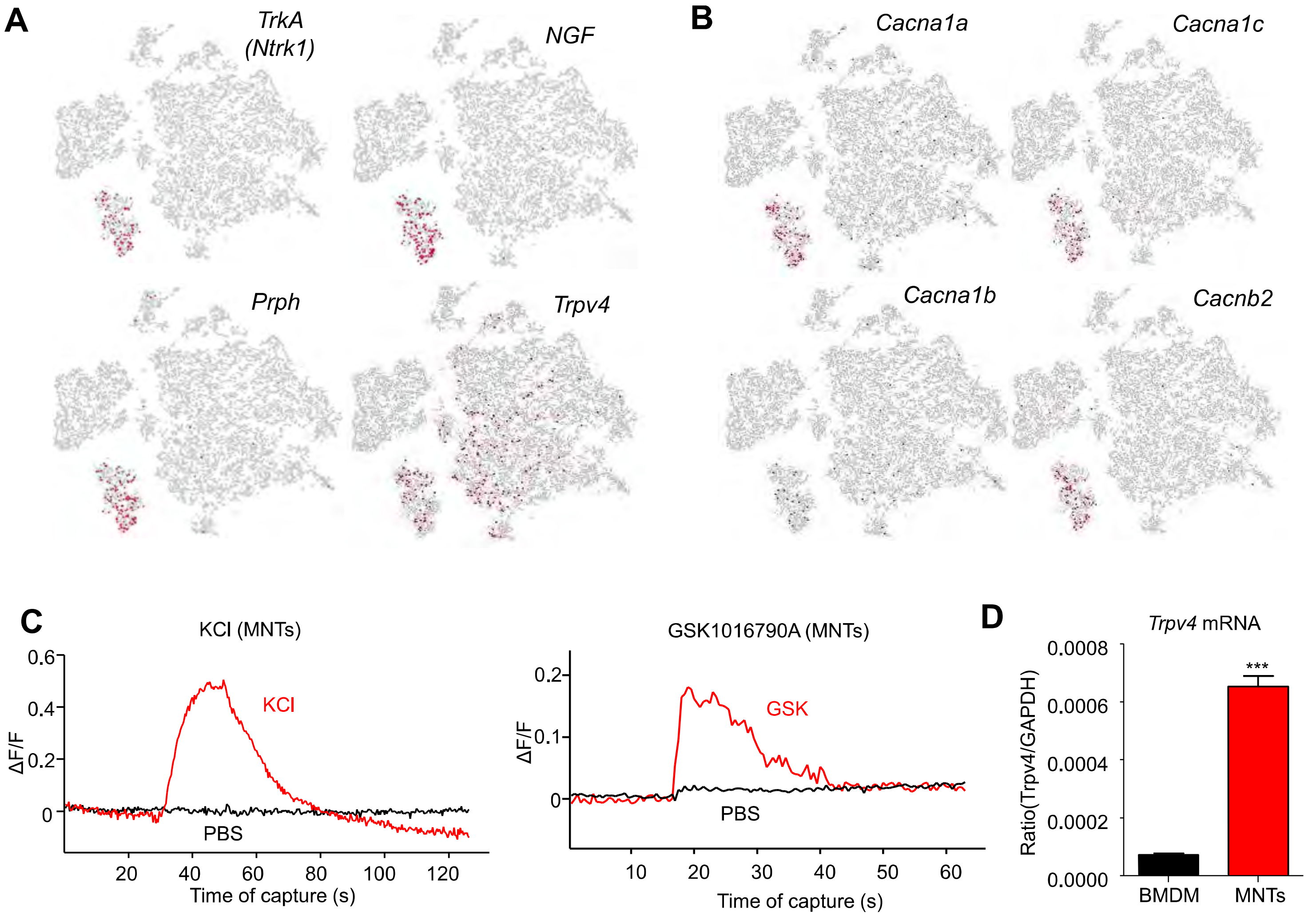

Fig. S10. Neuronal functional marker expression and phenotypes related to voltage dependent ion channels in MNTs *in vivo* and *in vitro*.

(A) Functional markers of sensory neuron and **(B)** voltage gated calcium channels are highly expressed in the MNTs *in vivo,* detecting by macrophage-lineage scRNA-seq analysis. **(C)** BMDM-derived MNTs respond to both KCl (20mM) and Trpv4 agonist (GSK1016790A, 2μM) *in vitro* showing by the neuronal calcium activity plots, where (**D**) Trpv4 is highly expressed in the MNTs detecting by real-time PCR (n=4, ***$p<0.001$ vs BMDM, t-test).

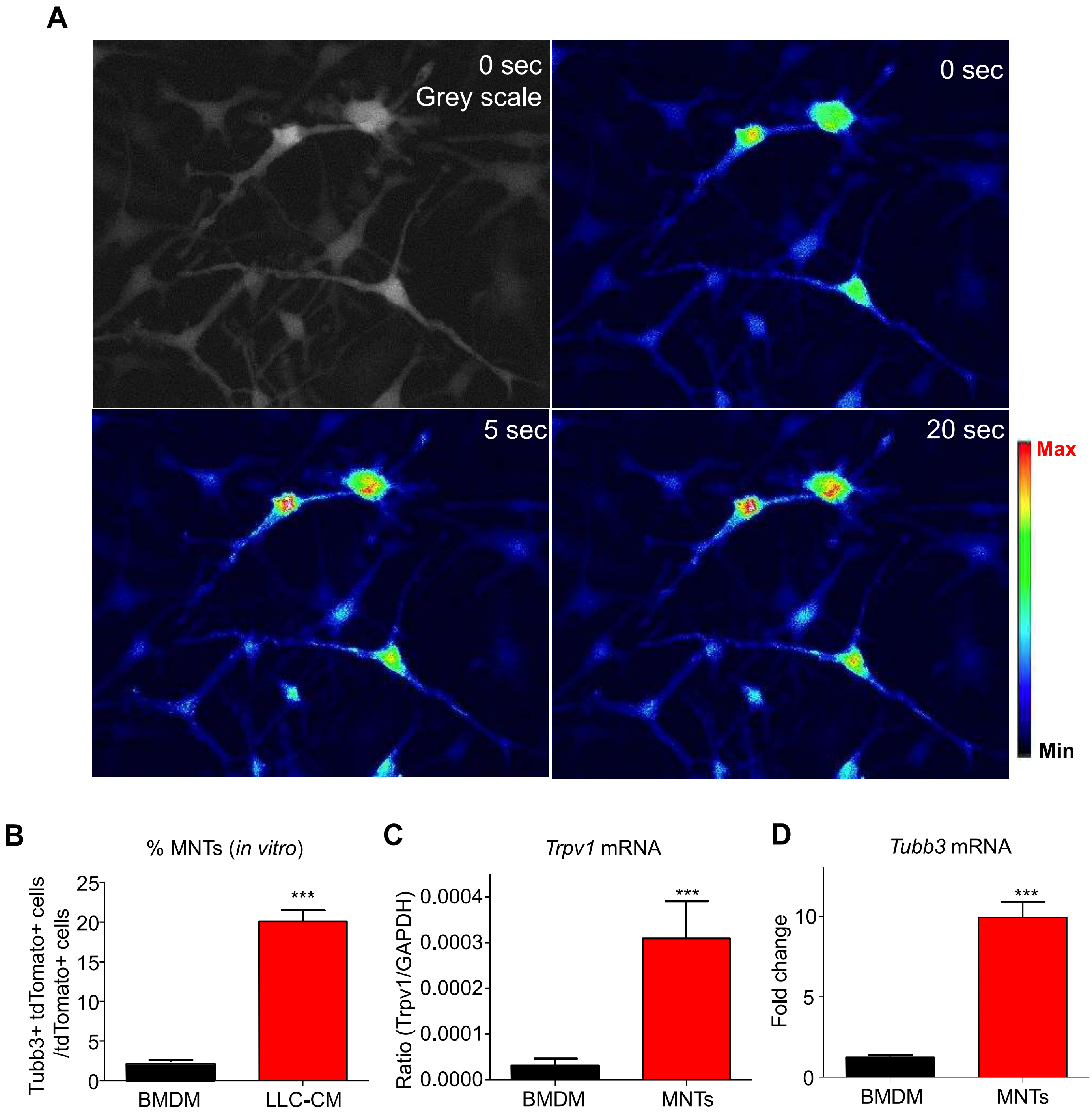

Fig. S11. Capsaicin-induced calcium efflux of BMDM-derived MNTs.

(A) Another representative image of BMDM-derived MNTs respond to the capsaicin induced calcium influx with grey scale and ratiometric format in a time course manner. Reproducibility of *in vitro* generation of MNTs, showing by **(B)** the abundance of tdTomato^{+} Tubb3^{+} cells in Fig. 4E, induction of **(C)** capsaicin receptor Trpv1 and **(D)** neuronal marker Tubb3 in the BMDM-derived MNTs *in vitro* (n=4, ***p<0.001 vs BMDM, t-test)

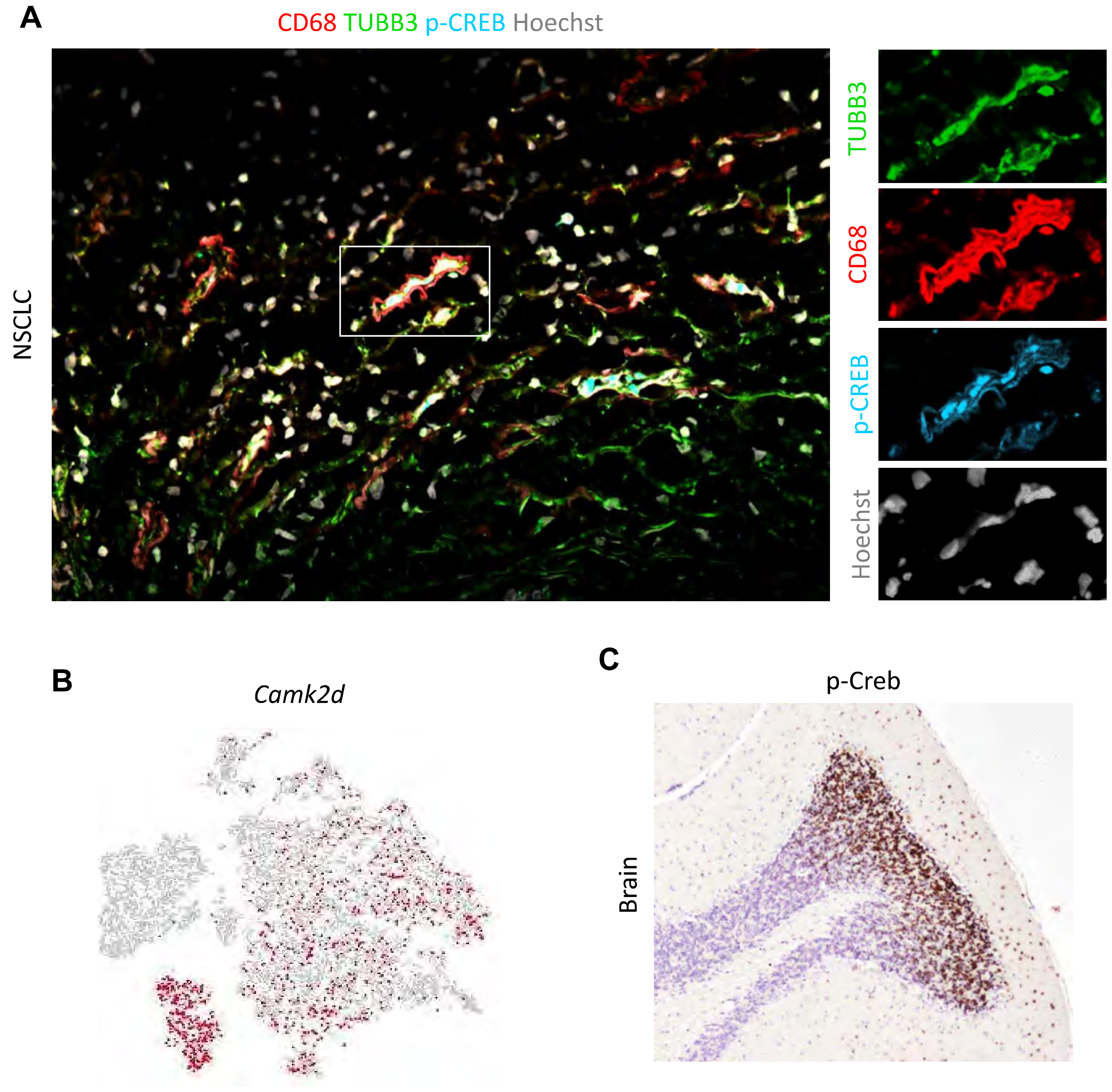

Fig. S12. Neuronal calcium signalling in human and mouse MNTs.

(A) Calcium activated neuronal transcription factor CREB is activated (p-CREB) in the human MNTs of a NSCLC biopsy detecting by confocal imaging. Consistently, (**B**) calcium dependent protein kinase of CREB (mouse gene: Camk2d) is highly expressed in the mouse MNTs detecting by macrophage-lineage scRNA-seq analysis. (**C**) Nuclear localization of p-Creb in cerebellar granule cells of a normal mouse brain is detected by IHC.

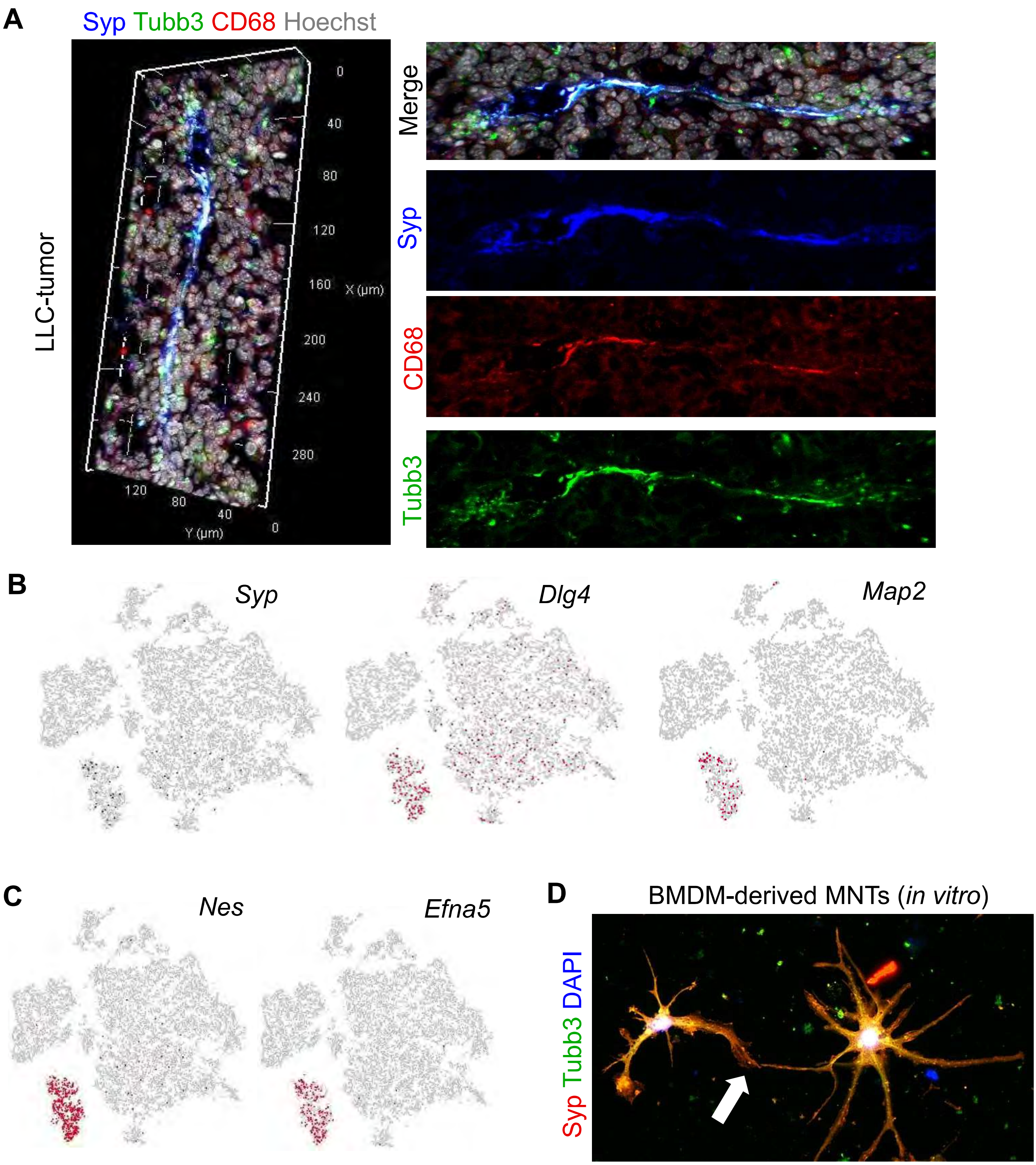

Fig. S13. Expression of synapse markers in MNTs *in vivo* and *in vitro*

(**A**) Confocal imaging revealed the presence of synapse markers synaptophysin (Syp) positive MNTs showing neuron like morphology in the LLC tumor. Macrophage lineage 10X scRNA-seq detected the expression of (**B**) synapse markers Syp, microtubule-associated protein 2 (Map2) and postsynaptic density protein 95 (PSD95/Dlg4) and (**C**) genes important for neuron development (Nes) and axon guidance (Efna5) in the MNTs *in vivo*. (**D**) Connection of Syp expressing BMDM-derived MNTs *in vitro* (white narrow) showing by immunofluorescence.

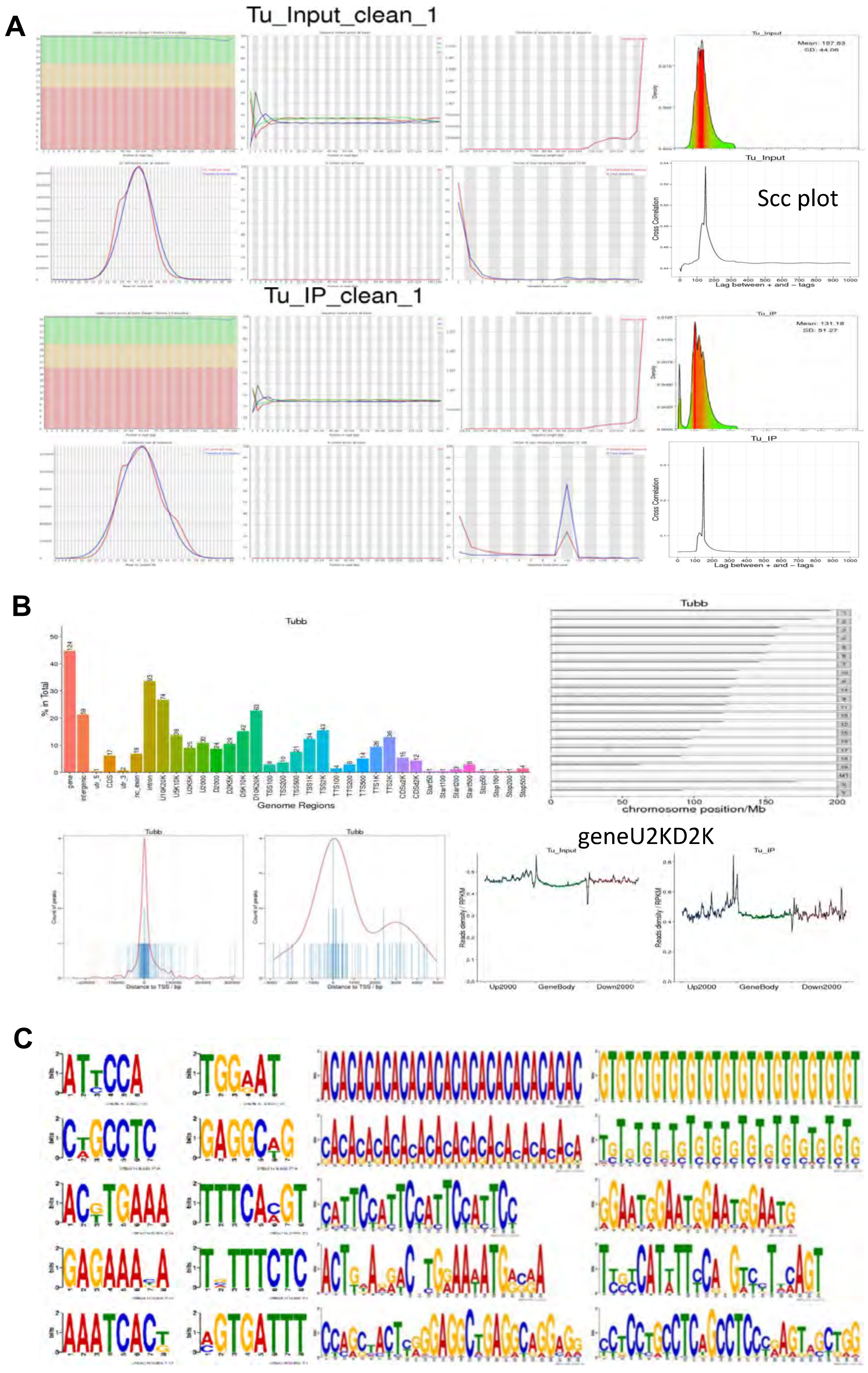

Fig. S14. ChIP-sequencing analysis results of Tubb3+ tdTomato+ MNT cells isolated from LLC tumor by FACS. (**A**) Quality control, (**B**) binding site distribution, (**C**) motif analysis.

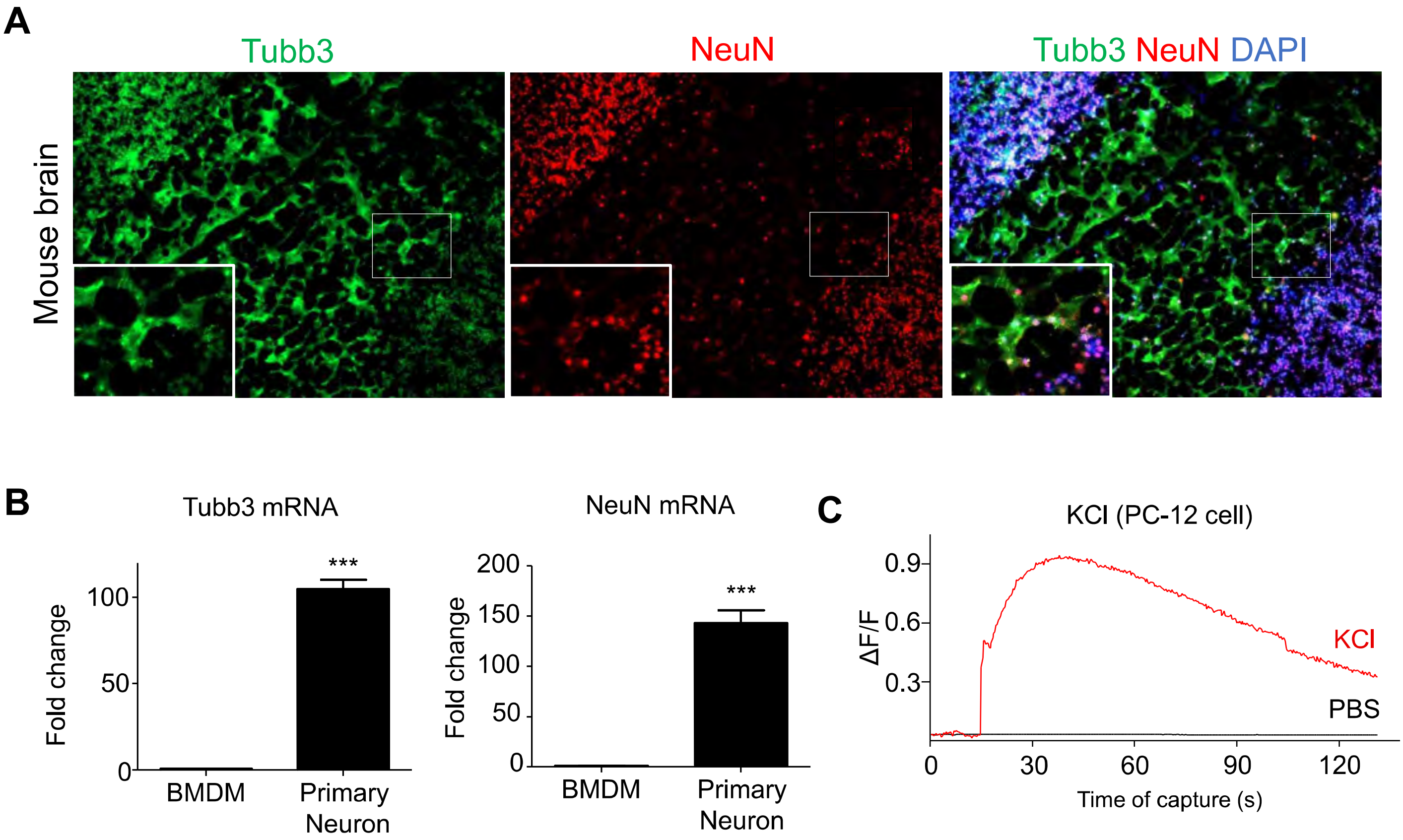

Fig. S15. Positive controls for neuronal phenotype validation.

(**A**) Classic neuronal markers Tubb3 and NeuN are highly expressed in a normal mouse brain. (**B**) Tubb3 and NeuN are highly expressed in mouse primary brain neurons compared to BMDM measuring by real-time PCR (n=4, ***$p<0.001$ vs BMDM, t-test). (**C**) KCl-induced calcium influx in neuronal cell line PC-12 detecting by OGB-1 assay.

Supplementary Note S1. Jupyter notebook for RNA velocity analysis

In [59]:
```
import scvelo as scv
import scanpy as sc
scv.logging.print_version()
import loompy
import sys
import numpy as np
import pandas as pd
import matplotlib
import loompy
import matplotlib.pyplot as plt
import scipy.optimize
# import velocyto as vcy
import glob
from sklearn.decomposition import PCA
from scipy.spatial.distance import pdist, squareform
import pickle
from IPython.core.display import display, HTML
display(HTML("<style>.container { width:90% !important; }</style>"))
%matplotlib inline
sc.settings.set_figure_params(dpi=100)
```

```
Running scvelo 0.2.2 (python 3.8.3) on 2020-11-18 00:55.
```

In [173]:
```
results_file = 'write/tomato.MNT.h5ad'
```

In [103]:
```
adata = sc.read_10x_mtx("./tomato/outs/filtered_feature_bc_matrix/",
var_names='gene_symbols',make_unique=True,cache=True)
```

In [104]:
```
adata_vel= scv.read('./tomato/velocyto/tomato.loom', cache=True)
adata = scv.utils.merge(adata, adata_vel)
```

```
Variable names are not unique. To make them unique, call `.var_names
_make_unique`.
```

In [105]:
```
adata.var_names_make_unique()
```

In [106]:
```
adata
```

Out[106]:
```
AnnData object with n_obs × n_vars = 11206 × 32285
    obs: 'Clusters', '_X', '_Y', 'initial_size_spliced', 'initial_si
ze_unspliced', 'initial_size'
    var: 'gene_ids', 'feature_types', 'Accession', 'Chromosome', 'En
d', 'Start', 'Strand'
    layers: 'ambiguous', 'matrix', 'spliced', 'unspliced'
```

Preprocessing

In [107]:
```
sc.pl.highest_expr_genes(adata, n_top=30)
```

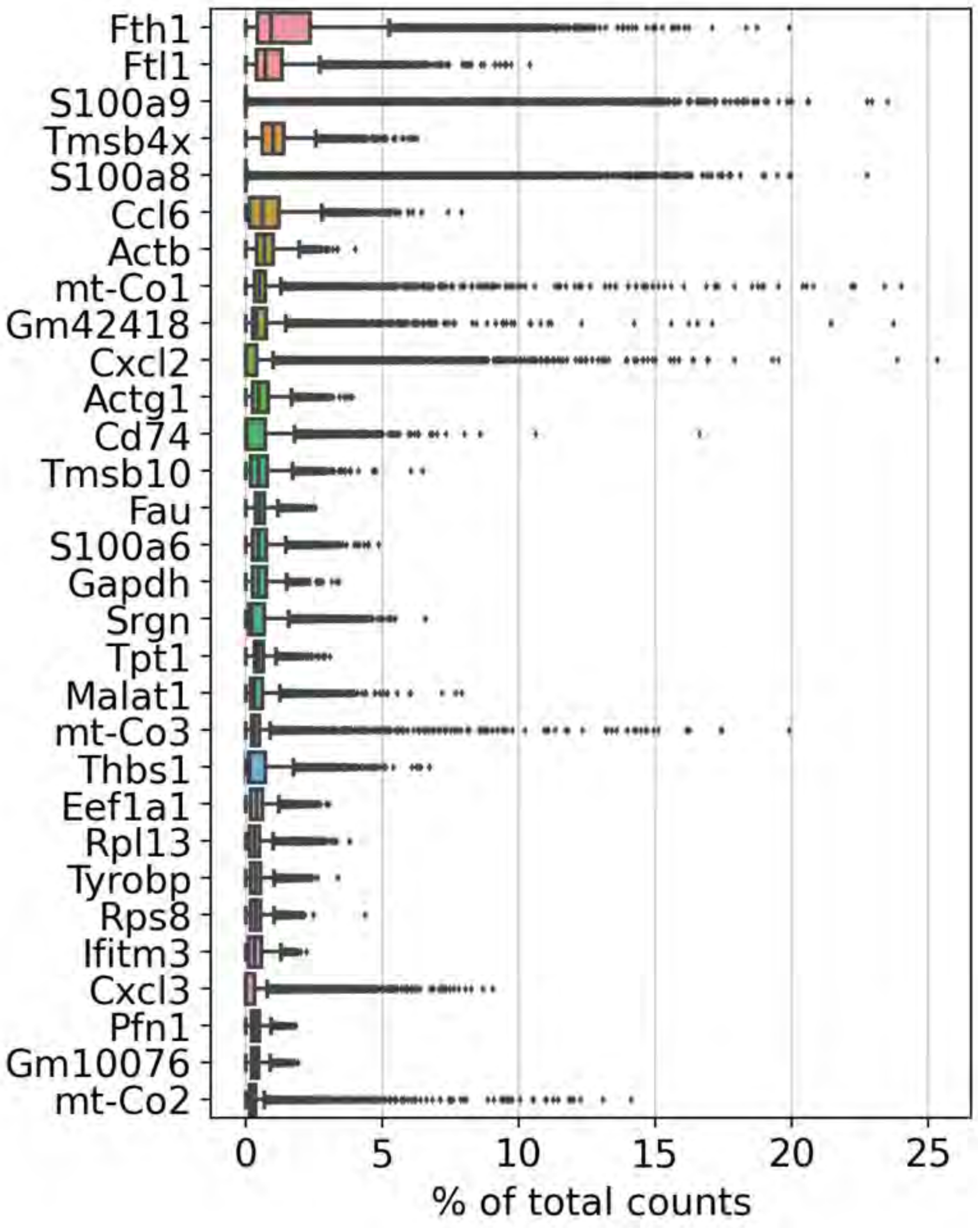

Basic filtering.

In [108]:
```
sc.pp.filter_cells(adata, min_genes=200)
sc.pp.filter_genes(adata, min_cells=3)
```

In [109]:
```
adata.var["mito"] = adata.var_names.str.startswith("mt-")
sc.pp.calculate_qc_metrics(adata, qc_vars=["mito"], inplace=True)
```

A violin plot of the computed quality measures.

In [110]:
```
sc.pl.violin(adata, ['n_genes_by_counts',
#                     'total_counts',
#                     'pct_counts_mito'
                    ],
             jitter=0.4, multi_panel=True)
sc.pl.violin(adata, [
#     'n_genes_by_counts',
                   'total_counts',
#                     'pct_counts_mito'
                    ],
             jitter=0.4, multi_panel=True)
sc.pl.violin(adata, [
#     'n_genes_by_counts',
#                     'total_counts',
                   'pct_counts_mito'
                    ],
             jitter=0.4, multi_panel=True)
```

```
... storing 'feature_types' as categorical
```

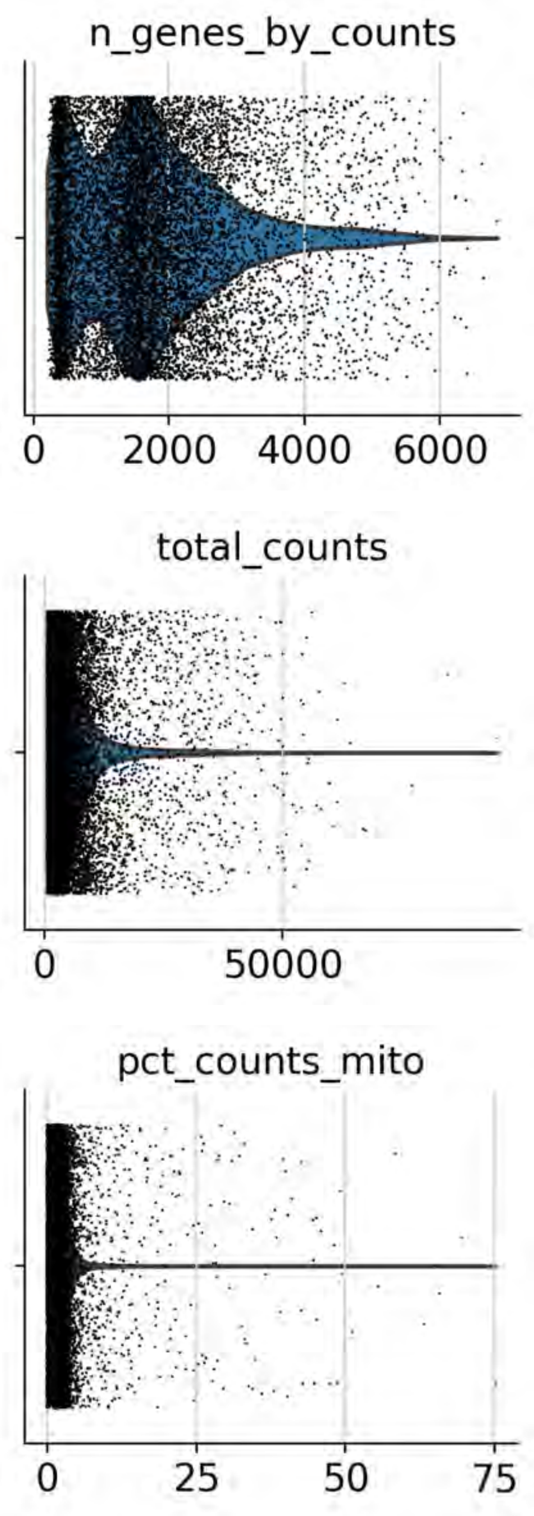

Remove cells that have too many mitochondrial genes expressed or too many total counts.

In [111]:
```
sc.pl.scatter(adata, x='total_counts', y='pct_counts_mito')
sc.pl.scatter(adata, x='total_counts', y='n_genes_by_counts')
```

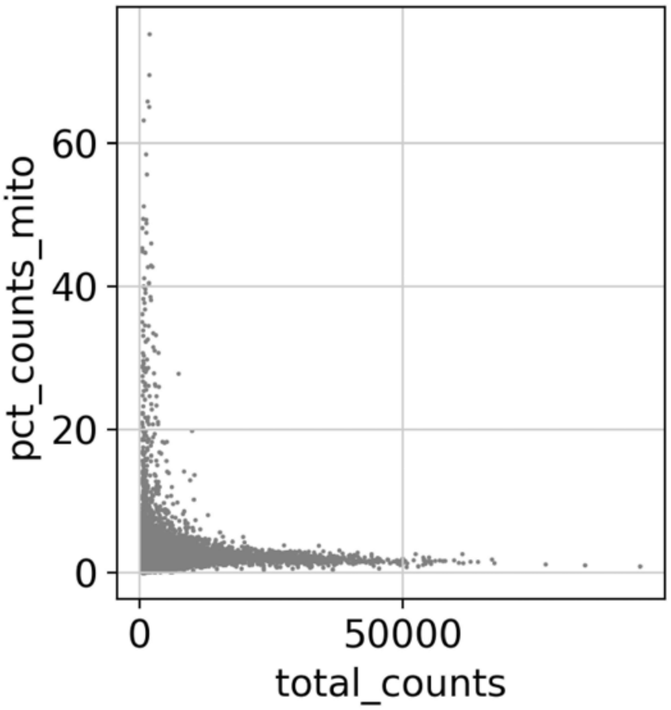

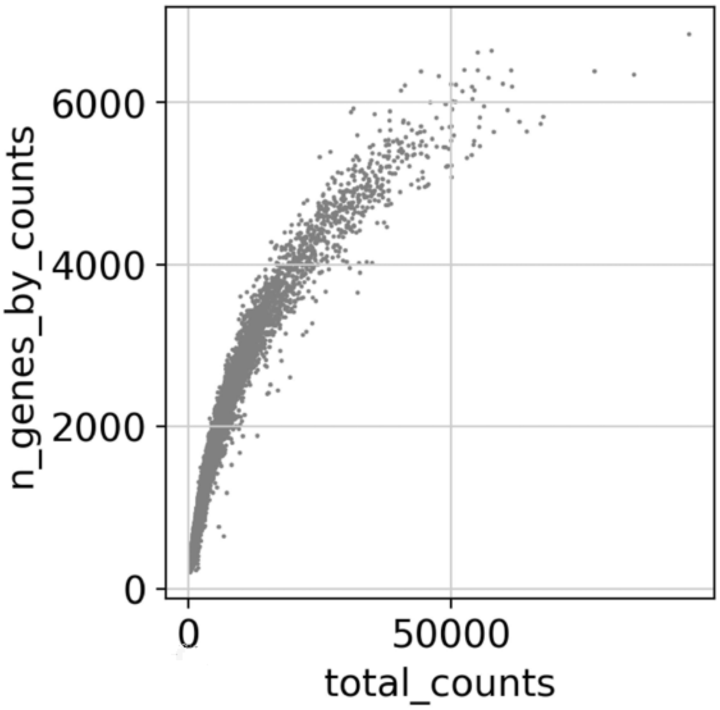

Actually do the filtering.

In [112]:
```
adata = adata[adata.obs['n_genes_by_counts'] < 5000, :]
adata = adata[adata.obs['pct_counts_mito'] < 10, :]
```

Set the `.raw` attribute of AnnData object to the logarithmized raw gene expression for later use in differential testing and visualizations of gene expression. This simply freezes the state of the AnnData object returned by `sc.pp.log1p`.

In [113]:
```
adata.raw = sc.pp.log1p(adata, copy=True)
```

Per-cell normalize the data matrix $\mathbf{X}$. Many people would consider the normalized data matrix as the "relevant data" for visualization and differential testing (assessing feature importance). Until a common viewpoint is reached on this, the decision of what to consider "raw", is up to the user. We tend to recommend to use the normalized data for visualization and differential testing even though here, we use the non-normalized data for the sake of consistency with the Seurat tutorial.

In [114]:
```
sc.pp.normalize_per_cell(adata, counts_per_cell_after=1e4)
```

Identify highly-variable genes.

In [176]:
```
adata.write(results_file)
```

In [116]:
```
filter_result = sc.pp.filter_genes_dispersion(
    adata.X, min_mean=0.1, max_mean=5, min_disp=0.25)
sc.pl.filter_genes_dispersion(filter_result)
```

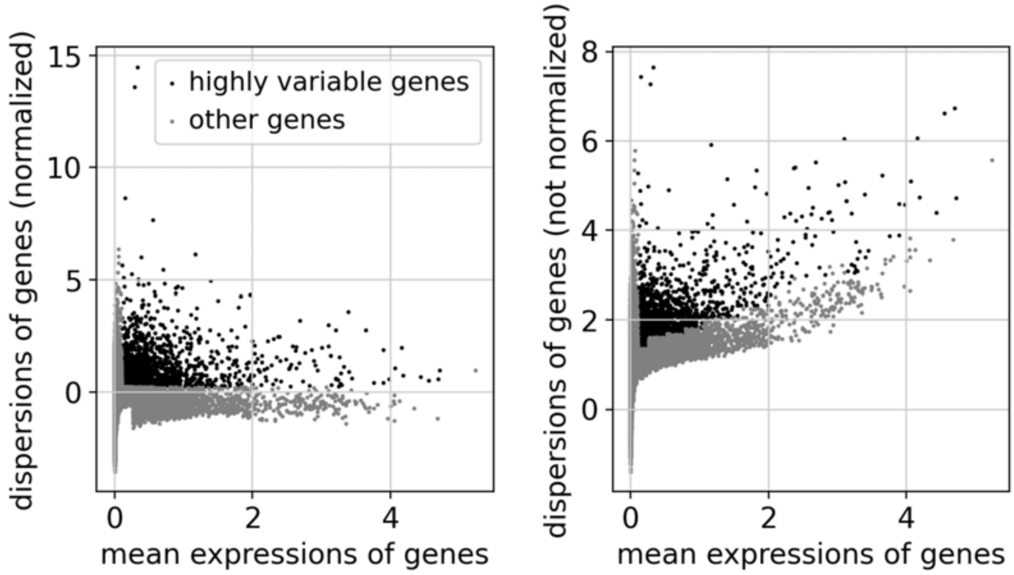

Actually do the filtering.

In [117]:
```
adata = adata[:, filter_result.gene_subset]
```

Logarithmize the data.

In [118]:
```
sc.pp.log1p(adata)
```

Regress out effects of total counts per cell and the percentage of mitochondrial genes expressed. Scale the data to unit variance.

In [119]:
```
sc.pp.regress_out(adata, ['total_counts','pct_counts_mito'])
```

Scale each gene to unit variance. Clip values exceeding standard deviation 10.

In [120]:
```
sc.pp.scale(adata, max_value=10)
```

Save the result.

In [121]:
```
adata.write(results_file)
```

In [122]:
```
adata
```

Out[122]:
```
AnnData object with n_obs × n_vars = 10766 × 2712
    obs: 'Clusters', '_X', '_Y', 'initial_size_spliced', 'initial_si
ze_unspliced', 'initial_size', 'n_genes', 'n_genes_by_counts', 'log1
p_n_genes_by_counts', 'total_counts', 'log1p_total_counts', 'pct_cou
nts_in_top_50_genes', 'pct_counts_in_top_100_genes', 'pct_counts_in_
top_200_genes', 'pct_counts_in_top_500_genes', 'total_counts_mito',
'log1p_total_counts_mito', 'pct_counts_mito', 'n_counts'
    var: 'gene_ids', 'feature_types', 'Accession', 'Chromosome', 'En
d', 'Start', 'Strand', 'n_cells', 'mito', 'n_cells_by_counts', 'mean
_counts', 'log1p_mean_counts', 'pct_dropout_by_counts', 'total_count
s', 'log1p_total_counts', 'mean', 'std'
    uns: 'log1p'
    layers: 'ambiguous', 'matrix', 'spliced', 'unspliced'
```

PCA

Compute PCA and make a scatter plot.

In [123]:
```
sc.tl.pca(adata, svd_solver='arpack')
```

Let us inspect the contribution of single PCs to the total variance in the data. This gives us information about how many PCs we should consider in order to compute the neighborhood relations of cells, e.g. used in the clustering function `sc.tl.louvain()` or tSNE `sc.tl.tsne()`. In our experience, often, a rough estimate of the number of PCs does fine. Seurat provides many more functions, here.

In [124]:
```
sc.pl.pca_variance_ratio(adata, log=True)
```

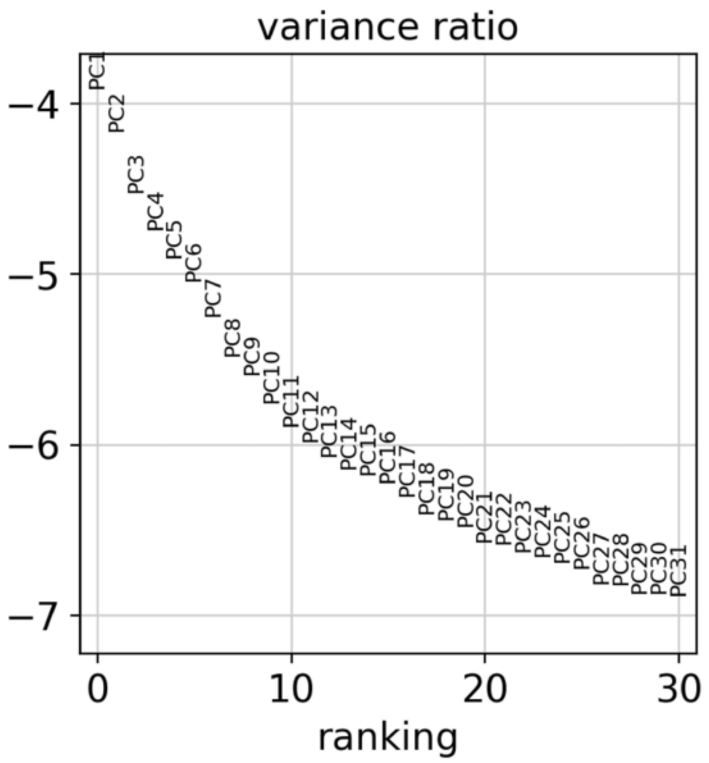

In [125]:
```
adata.write(results_file)
```

In [126]:
```
adata
```

Out[126]:
```
AnnData object with n_obs × n_vars = 10766 × 2712
    obs: 'Clusters', '_X', '_Y', 'initial_size_spliced', 'initial_si
ze_unspliced', 'initial_size', 'n_genes', 'n_genes_by_counts', 'log1
p_n_genes_by_counts', 'total_counts', 'log1p_total_counts', 'pct_cou
nts_in_top_50_genes', 'pct_counts_in_top_100_genes', 'pct_counts_in_
top_200_genes', 'pct_counts_in_top_500_genes', 'total_counts_mito',
'log1p_total_counts_mito', 'pct_counts_mito', 'n_counts'
    var: 'gene_ids', 'feature_types', 'Accession', 'Chromosome', 'En
d', 'Start', 'Strand', 'n_cells', 'mito', 'n_cells_by_counts', 'mean
_counts', 'log1p_mean_counts', 'pct_dropout_by_counts', 'total_count
s', 'log1p_total_counts', 'mean', 'std'
    uns: 'log1p', 'pca'
    obsm: 'X_pca'
    varm: 'PCs'
    layers: 'ambiguous', 'matrix', 'spliced', 'unspliced'
```

Computing the neighborhood graph

In [177]:
```
adata = sc.read(results_file)
```

Let us compute the neighborhood graph of cells using the PCA representation of the data matrix. You might simply use default values here. For the sake of reproducing Seurat's results, let's take the following values.

In [128]:
```
sc.pp.neighbors(adata, n_neighbors=20, n_pcs=40)
```

We now advertise visualizing the data using UMAP, see below. In particular, if you have large data, this will give you a notable speedup. Also, it is potentially more faithful to global topology: trajectories are better preserved.

In [129]:
```
sc.tl.umap(adata)
```

Clustering the graph

As Seurat and many others, we recommend the Louvain graph-clustering method (community detection based on optimizing modularity). It has been proposed for single-cell data by [Levine *et al.* (2015) (https://doi.org/10.1016/j.cell.2015.05.047)](https://doi.org/10.1016/j.cell.2015.05.047). Note that Louvain clustering directly clusters the neighborhood graph of cells, which we already computed in the previous section.

In [130]:
```
sc.tl.leiden(adata,resolution=0.3)
```

Plot the data with tSNE. Coloring according to clustering. Clusters agree quite well with the result of Seurat.

In [181]:
```
sc.pl.umap(adata, color=['leiden','Adgre1',"Cd68","Tubb3"])
```

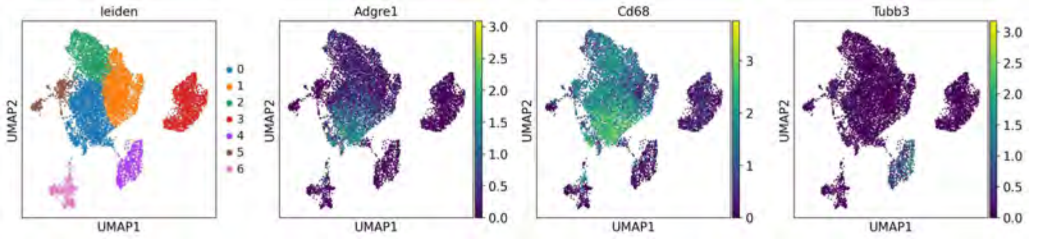

In [132]:
```
sc.tl.paga(adata)
sc.pl.paga(adata, plot=False)  # remove `plot=False` if you want to
 see the coarse-grained graph
sc.tl.umap(adata, init_pos='paga')
```

In [134]:
```
sc.pl.paga(adata, color=['leiden'])
```

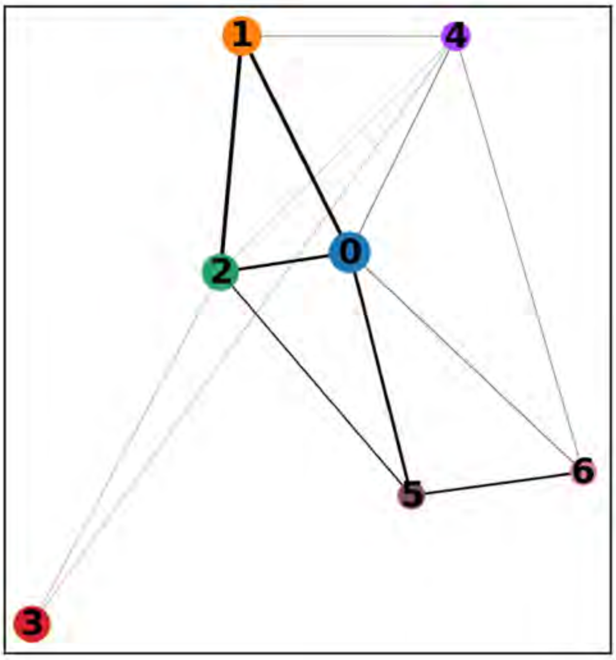

In [135]:
```
sc.tl.draw_graph(adata, init_pos='paga')
```

In [180]:
```
sc.pl.draw_graph(adata, color=['leiden','Tubb3', 'Adgre1',"Cd68","Rp
l13","Nedd4"], legend_loc='on data')
```

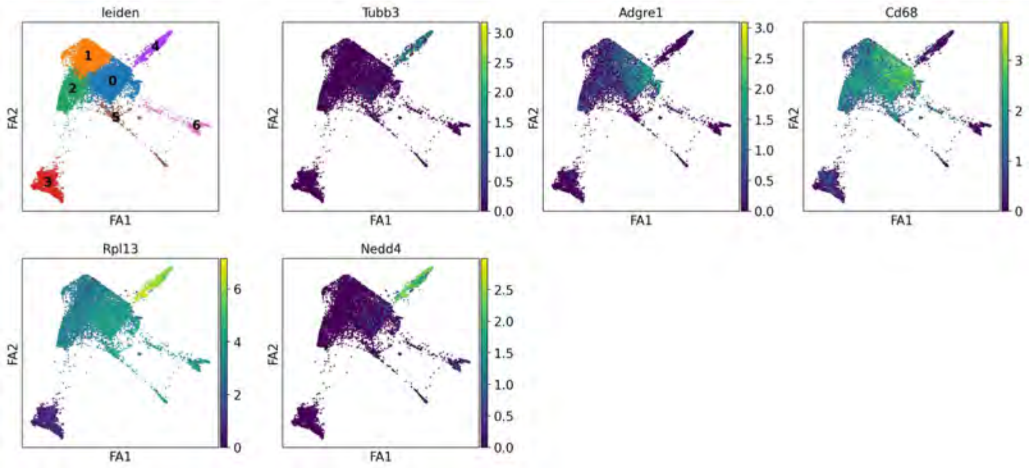

In [138]:
```
adata.uns['iroot'] = np.flatnonzero(adata.obs['leiden']  == '0')[0]
```

In [139]:
```
sc.tl.dpt(adata)
```

```
WARNING: Trying to run `tl.dpt` without prior call of `tl.diffmap`.
Falling back to `tl.diffmap` with default parameters.
```

In [182]:
```
sc.pl.draw_graph(adata, color=['leiden', 'dpt_pseudotime'], legend_loc='on data')
```

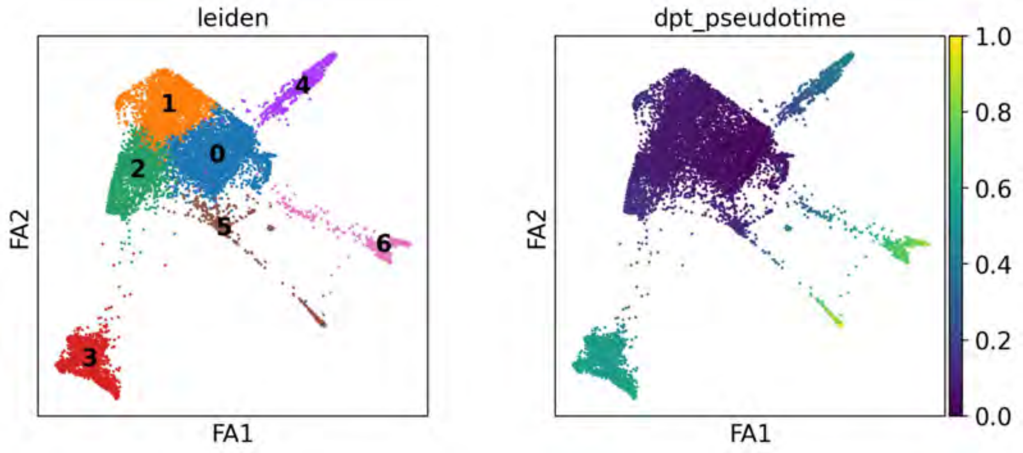

In [144]:
```
sc.tl.rank_genes_groups(adata, 'leiden')
sc.pl.rank_genes_groups(adata, n_genes=20, sharey=False)
```

```
WARNING: Default of the method has been changed to 't-test' from 't-test_overestim_var'
```

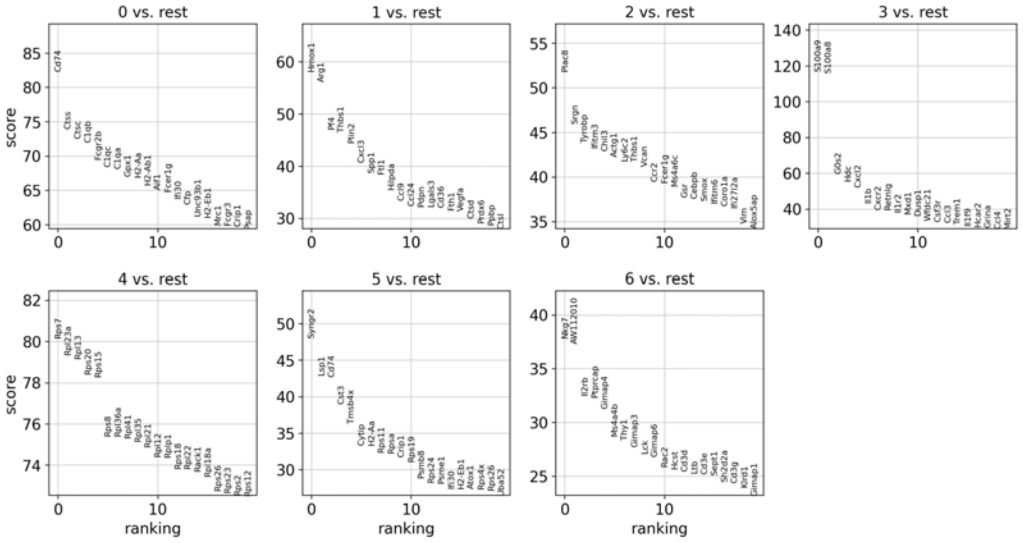

In [172]:
```
pd.DataFrame(adata.uns['rank_genes_groups']['names']).to_csv("tomato.adata.marker.csv")
```

add scvelo

In [146]:
```
scv.pp.filter_genes(adata, min_shared_counts=10)
scv.pp.normalize_per_cell(adata)
scv.pp.filter_genes_dispersion(adata, n_top_genes=3000)
scv.pp.log1p(adata)
```

```
Filtered out 299 genes that are detected 10 counts (shared).
WARNING: Did not normalize X as it looks processed already. To enfor
ce normalization, set `enforce=True`.
Normalized count data: spliced, unspliced.
Skip filtering by dispersion since number of variables are less than
`n_top_genes`.
```

In [147]:
```
scv.pp.filter_and_normalize(adata, min_shared_counts=30, n_top_genes
=2000)
scv.pp.moments(adata, n_pcs=30, n_neighbors=30)
```

```
Filtered out 606 genes that are detected 30 counts (shared).
WARNING: Did not normalize X as it looks processed already. To enfor
ce normalization, set `enforce=True`.
WARNING: Did not normalize spliced as it looks processed already. To
enforce normalization, set `enforce=True`.
WARNING: Did not normalize unspliced as it looks processed already.
To enforce normalization, set `enforce=True`.
Skip filtering by dispersion since number of variables are less than
`n_top_genes`.
WARNING: Did not modify X as it looks preprocessed already.
computing neighbors
    finished (0:00:06) --> added
    'distances' and 'connectivities', weighted adjacency matrices (a
data.obsp)
computing moments based on connectivities
    finished (0:00:01) --> added
    'Ms' and 'Mu', moments of un/spliced abundances (adata.layers)
```

In [148]:
```
adata
```

Out[148]:
```
AnnData object with n_obs × n_vars = 10766 × 1807
    obs: 'Clusters', '_X', '_Y', 'initial_size_spliced', 'initial_si
ze_unspliced', 'initial_size', 'n_genes', 'n_genes_by_counts', 'log1
p_n_genes_by_counts', 'total_counts', 'log1p_total_counts', 'pct_cou
nts_in_top_50_genes', 'pct_counts_in_top_100_genes', 'pct_counts_in_
top_200_genes', 'pct_counts_in_top_500_genes', 'total_counts_mito',
'log1p_total_counts_mito', 'pct_counts_mito', 'n_counts', 'leiden',
'dpt_pseudotime'
    var: 'gene_ids', 'feature_types', 'Accession', 'Chromosome', 'En
d', 'Start', 'Strand', 'n_cells', 'mito', 'n_cells_by_counts', 'mean
_counts', 'log1p_mean_counts', 'pct_dropout_by_counts', 'total_count
s', 'log1p_total_counts', 'mean', 'std'
    uns: 'log1p', 'pca', 'neighbors', 'umap', 'leiden', 'leiden_colo
rs', 'paga', 'leiden_sizes', 'draw_graph', 'iroot', 'diffmap_evals',
'rank_genes_groups'
    obsm: 'X_pca', 'X_umap', 'X_draw_graph_fa', 'X_diffmap'
    varm: 'PCs'
    layers: 'ambiguous', 'matrix', 'spliced', 'unspliced', 'Ms', 'M
u'
    obsp: 'distances', 'connectivities'
```

In [183]:
```
scv.tl.velocity(adata)
scv.tl.velocity_graph(adata)
scv.pl.velocity_embedding_stream(adata, basis='X_draw_graph_fa',colo
r = 'leiden' )
```

```
computing velocities
    finished (0:00:02) --> added
    'velocity', velocity vectors for each individual cell (adata.lay
ers)
computing velocity graph
    finished (0:00:29) --> added
    'velocity_graph', sparse matrix with cosine correlations (adata.
uns)
computing velocity embedding
    finished (0:00:02) --> added
    'velocity_draw_graph_fa', embedded velocity vectors (adata.obsm)
```

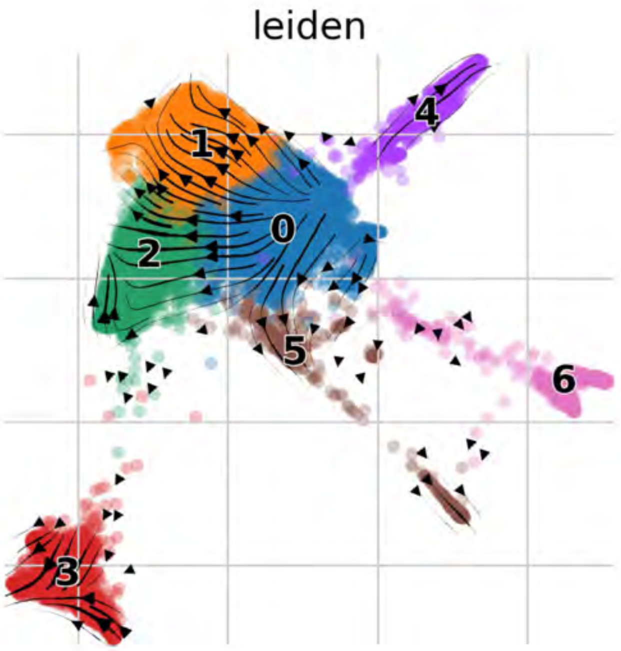

In [184]:
```
scv.pl.velocity_embedding(adata, color = "leiden",basis='X_draw_graph_fa', arrow_length=4, arrow_size=4, dpi=300)
```

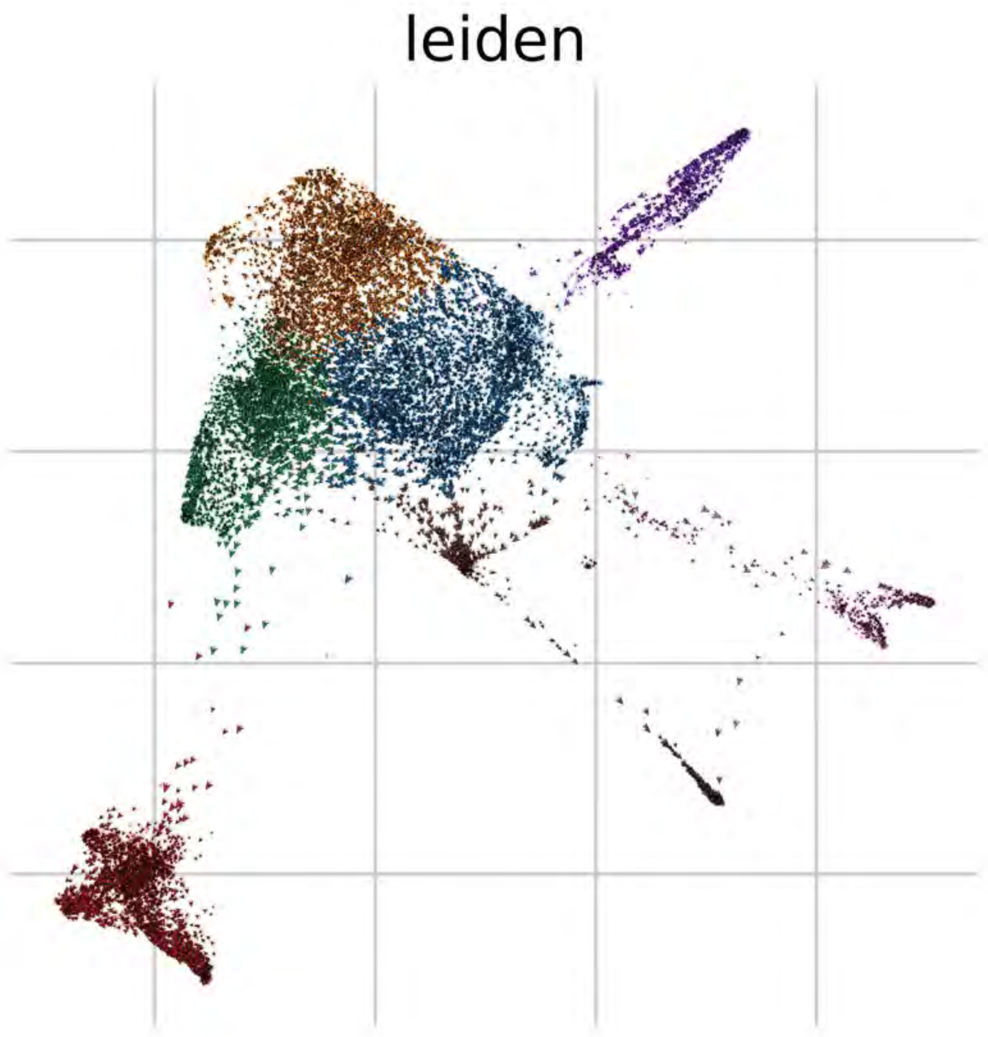

In [156]:
```
adata.uns['neighbors']['distances'] = adata.obsp['distances']
adata.uns['neighbors']['connectivities'] = adata.obsp['connectivitie
s']

scv.tl.paga(adata, groups='leiden')
df = scv.get_df(adata, 'paga/transitions_confidence', precision=2).T
df.style.background_gradient(cmap='Blues').format('{:.2g}')
```

```
running PAGA using priors: ['velocity_pseudotime']
    finished (0:00:02) --> added
    'paga/connectivities', connectivities adjacency (adata.uns)
    'paga/connectivities_tree', connectivities subtree (adata.uns)
    'paga/transitions_confidence', velocity transitions (adata.uns)
```

Out[156]:

	0	1	2	3	4	5	6
**0**	0	0.12	0.15	0	0	0.12	0
**1**	0	0	0	0	0	0	0
**2**	0	0	0	0	0	0	0
**3**	0	0	0	0	0	0	0
**4**	0.037	0	0	0.018	0	0	0
**5**	0	0	0	0	0	0	0
**6**	0	0	0	0	0	0.057	0

In [160]:
```
scv.pl.paga(adata, basis='draw_graph_fa', size=50, alpha=.1,
            min_edge_width=2, node_size_scale=1.5)
```

```
WARNING: Invalid color key. Using grey instead.
```

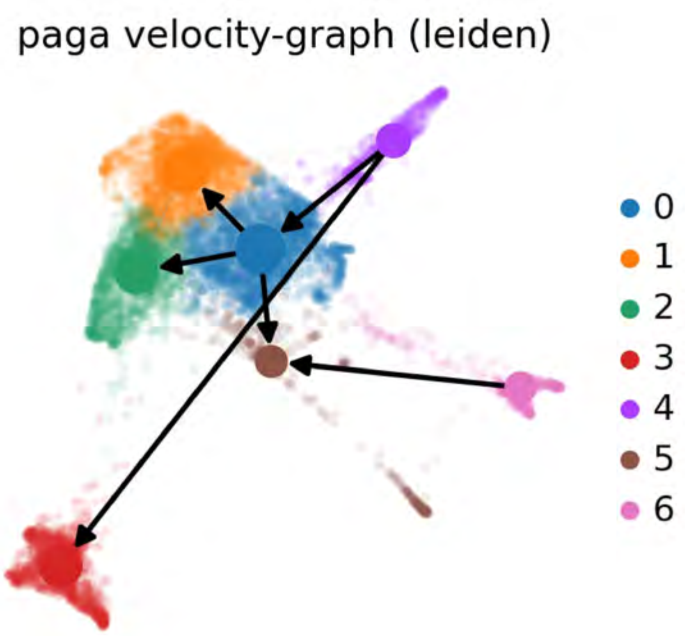

In [ ]:

In [ ]:

```

```

In [161]:

```
scv.tl.rank_velocity_genes(adata, groupby='leiden')
scv.DataFrame(adata.uns['rank_velocity_genes']['names']).head(n=10)
```

```
ranking velocity genes
    finished (0:00:04) --> added
    'rank_velocity_genes', sorted scores by group ids (adata.uns)
    'spearmans_score', spearmans correlation scores (adata.var)
```

Out[161]:

	0	1	2	3	4	5	6
**0**	Frmd4b	Cacna1d	Ifitm6	4732465J04Rik	Wdr89	Cst3	Ptpn22
**1**	Clec12a	Flrt2	Socs3	Alcam	Sparc	Cytip	Cblb
**2**	Slc9a9	Plin2	Trps1	Slc7a11	Fscn1	P2ry10	Cd37
**3**	Dhrs3	Stab1	Cytip	Msra	Ptges	Grk3	Rabgap1l
**4**	H2-Ab1	Osbpl8	Emilin2	Sik3	AU020206	Map3k14	Ptprc
**5**	Pkib	Rusc2	Thbs1	Cass4	Actn1	Lsp1	D16Ertd472e
**6**	Stard8	Fnip2	Havcr2	Myo1d	Hmgb2	Mrtfa	Akap13
**7**	Dapk1	Katnbl1	Ms4a4c	Il1rap	Socs1	Cd83	Traf3ip3
**8**	AB124611	Pitpnc1	Vcan	Nedd9	Creg1	H2-Ab1	Gimap4
**9**	Ms4a4a	Abca1	Dok3	Lilr4b	Mt2	Ifitm1	Fryl

In [165]:

```
scv.pl.velocity(adata, ['Wdr89', 'Sparc', 'Creg1', 'Actn1'],color='l
eiden', ncols=2)
```

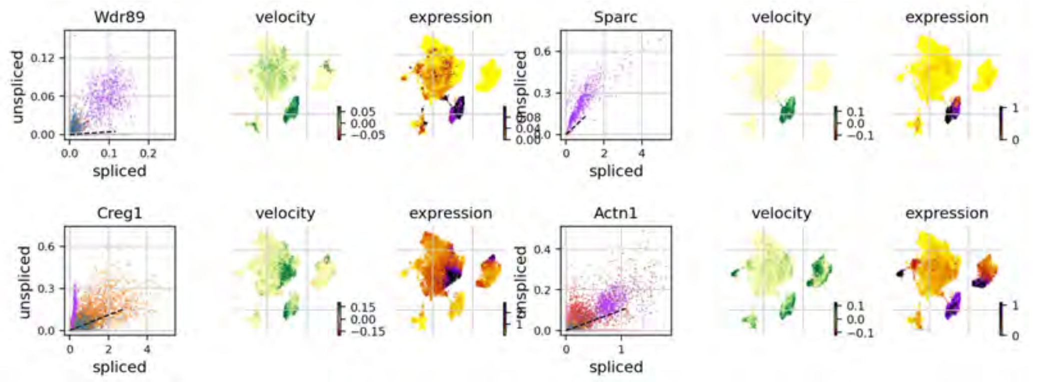

Supplementary Note S2. R script for pseudotime analysis.

```
#Input data
library(monocle)
expr_matrix <- read.csv("count.csv",header=1, row.names=1)
expr_matrix <- as.matrix(expr_matrix)
sample_sheet <- read.csv("barcode.csv", header=1,row.names=1)
gene_annotation <- read.csv("gene.csv",header=1,row.names=1)
#Store Data in a CellDataSet Object
pd <- new("AnnotatedDataFrame", data = sample_sheet)
fd <- new("AnnotatedDataFrame", data = gene_annotation)
colnames(pd) <- c("cell")
colnames(fd) <- c("gene_short_name")
rownames(pd) <- colnames(expr_matrix)
rownames(fd) <- rownames(expr_matrix)
cds <- newCellDataSet(expr_matrix, phenoData = pd, featureData = fd)
#Estimate size factors and dispersions
cds <- estimateSizeFactors(cds)
cds <- estimateDispersions(cds)
#Filtering low-quality cells
#cds <- detectGenes(cds, min_expr = 0.1)
#expressed_genes <- row.names(subset(fData(cds),
    #num_cells_expressed >= 10))
#print(head(fData(cds)))
#Classify cells with known marker genes
cth <- newCellTypeHierarchy()
Tubb3_id <- row.names(subset(fData(cds), gene_short_name == "Tubb3"))
Cd68_id <- row.names(subset(fData(cds), gene_short_name == "Cd68")) cth
<- addCellType(cth, "Tubb3+Cd68-", classify_func = function(x)
{ x[Tubb3_id,] > 0 & x[Cd68_id,] <= 0 })
cth <- addCellType(cth, "Tubb3-Cd68+", classify_func = function(x)
{ x[Cd68_id,] > 0 & x[Tubb3_id,] <= 0 })
cth <- addCellType(cth, "Tubb3+Cd68+", classify_func = function(x)
{ x[Tubb3_id,] > 0 & x[Cd68_id,] > 0 })
cth <- addCellType(cth, "Tubb3-Cd68-", classify_func = function(x)
{ x[Tubb3_id,] < 0 & x[Cd68_id,] < 0 })
cds <- classifyCells(cds, cth, 0.1)
table(pData(cds)$CellType)
cds <- reduceDimension(cds, max_components = 2, num_dim = 6,
                reduction_method = 'tSNE')
cds <- clusterCells(cds)
plot_cell_clusters(cds, 1, 2, cell_size = 1,color_by = "CellType") cds
<- reduceDimension(cds, max_components = 4,method = 'DDRTree') cds <-
orderCells(cds)
plot_cell_trajectory(cds, color_by = "CellType")
plot_cell_trajectory(cds, color_by = "Pseudotime")
```

Target	Concentration	Catalogue number	Manufacturer	Application
GAPDH	1:1000	sc-32233	Santa Cruz	Western blotting
NeuN	1:1000	sc-246957	Santa Cruz	
Pou4f1	1:1000	sc-8429	Santa Cruz	
Tuj-1	1:1000	sc-5274	Santa Cruz	
F4/80	1:100	123110 (PE)<br>123108 (FITC)	Biolegend	Immunofluorescent staining<br>Flow cytometry<br>Opal multiplexing
CD68	1:100	sc-20060	Santa Cruz	
Tubb3	1:100	sc-74412	Santa Cruz	
NeuN	1:1000	sc-246957	Santa Cruz	
p-Smad3	1:200	600-401-919	Rockland	
Syp	1:200	S5768	Sigma	
p-Creb	1:500	9198S	Cell signaling technology	
Smad3	1:100	9523s	Cell signaling technology	ChIP assay and ChIP sequceing
IgG Isotype	1:1000	3900S	Cell signaling technology	

Table S1. Antibodies used in this study.

	Forward primer (5' to 3')	Reverse primer (5' to 3')
Gapdh	GCATGGCCTTCCGTGTTC	GATGTCATCATACTTGGCAGGTTT
Tubb3	AGTCAGCATGAGGGAGATCG	AGTCCCCTACATAGTTGCCG
NeuN	CAGACAACCAGCAACTCCAC	CGAATTGCCCGAACATTTGC
Trpv1	GAGGACCCAGGCAACTGTGA	CTCCCTGAAACTCGGCCTGA
Trpv4	ATGGCAGATCCTGGTGATGG	GGAACTTCATACGCAGGTTTGG
ChIP-Smad3-Tubb3	GGTGCTGATGCTGCAGAG	CTGGGCTCTTCCCTTTGTTC

Table S2. List of primers used in this study

Number detected in scRNA-seq	Human NSCLC	Mouse macrophage lineage
Source	Fresh surgical resection of a non-small cell lung carcinoma tumor.	FACS tdTomato cells from LLC tumour on LysM-tdTomato mice<br>(pooled from 8 tumors)
Total cells detected	7,802	11,196
Total reads	376,028,493	527,277,911
Total Genes	23,148	18,583
Mean Reads per Cell	48,196	47,095
Median Genes per Cell	1,442	1,547
Median UMI Counts per Cell	4,197	3,962
Valid barcodes	92.9%	96.9%

Table S3. Quality control statistics of scRNA-seq datasets

Other Supplementary Materials

File S1

Metacore enrichment analysis of DEGs extracted from $Tubb3^+$ cluster in macrophage lineage tdTomato cells.

File S2

Disease association of up-regulated DEGs of $TUBB3^+$ and $TUBB3^+$ $CD68^+$ cells in NSCLC.

File S3

GO analysis of ChIP-seq detected Smad3 direct genes in the $Tubb3^+$ MNTs.

Video S1

Confocal live imaging detected some $tdTomato^+$ macrophage-lineage cells displaying nerve fibre-like morphology in the LLC-tumor, visualizing their OGB-1 retention *ex vivo*.

Video S2

Live imaging captured the nociceptive activity of BMDM-derived MNTs upon capsaicin stimulation, showing the OGB-1 efflux *in vitro*.
